# Neuroprotective effects of traditional Chinese medicine formulas in animal models of retinal degenerative diseases: a systematic review and meta-analysis

**DOI:** 10.3389/fphar.2025.1695150

**Published:** 2026-01-07

**Authors:** Yunxi Xu, Qindong Mi, Qi Yong, Chao Xu, Dingmeng Zhao, Chuning Wang, Zhongshan Jiang, Chenghao Yu, Hejiang Ye

**Affiliations:** 1 School of Ophthalmology, Chengdu University of Traditional Chinese Medicine, Chengdu, China; 2 Department of Ophthalmology, Hospital of Chengdu University of Traditional Chinese Medicine, Chengdu, Sichuan, China; 3 Department of Traditional Chinese Medicine, Chinese Academy of Medical Sciences and Peking Union Medical College, Beijing, China; 4 School of Basic Medical Sciences, Chengdu University of Traditional Chinese Medicine, Chengdu, Sichuan, China

**Keywords:** neuroprotection, traditional Chinese medicine, apoptosis, oxidative stress, meta-analysis, retinal degenerative diseases

## Abstract

**Purpose:**

Retinal degenerative diseases (RDDs) cause irreversible vision loss with limited treatment options. Traditional Chinese medicine (TCM) formulas have demonstrated neuroprotective effects, yet their overall efficacy lacks comprehensive meta-evidence. The aim of this study was to exploratively evaluate the neuroprotective effects of TCM formulas in animal RDD models.

**Methods:**

A comprehensive literature search was conducted across eight electronic databases to identify animal studies that evaluated the neuroprotective effects of TCM formulas on RDDs. Pairwise meta-analysis and Bayesian network meta-analysis (NMA) were performed to synthesize evidence on key outcomes: neural growth, glial activation, oxidative stress, apoptosis factors, and ophthalmological parameters. Treatment rankings were assessed using the surface under the cumulative ranking curve (SUCRA).

**Results:**

Twenty-four studies were included. The compositions and bioactive compounds of the TCM formulas have been defined and identified. Pairwise meta-analysis demonstrated that specific TCM formulas might exert neuroprotective effects on RDDs by regulating key biomarkers. Specifically, Zhen-Bao-Wan, Bu-Shen-Yi-Jing-Fang, and Qi-Shen-Yi-Qi pills modulated neural growth and glial activation by upregulating BDNF, CNTF, and reducing GFAP, respectively. Furthermore, Yi-Qi-Wen-Yang-Tong-Luo decoction, Zi-Yin-Ming-Mu decoction, and Yishi-Tablet suppressed oxidative stress and apoptosis by reducing SOD, retinal apoptotic cells and caspase-3, respectively. Additionally, Bu-Yang-Huan-Wu decoction improved retinal function by elevating ERG-a and ERG-b wave amplitudes. Subgroup analyses indicated that Bu-Yang-Huan-Wu decoction and Qu-Yu-Tong-Luo prescription exhibited superior efficacy in restoring retinal ganglion cell (RGC) counts and retinal thickness in specific RDD models. The NMA results indicated that the included TCM formulas exhibited target-specific and dose‒response trends, with different formulas showing preferential efficacy for distinct biomarkers. Given the limitations identified in this study, these findings should be interpreted as preliminary evidence to guide future research rather than as conclusive results. Future studies with rigorous experimental designs are needed to address these limitations and enhance translational relevance.

**Conclusion:**

This study provides preclinical and exploratory evidence that the included TCM formulas might exert neuroprotective effects on animal models of RDDs by modulating glial activation, promoting neuronal growth, and inhibiting oxidative stress and apoptosis. Additional high-quality preclinical studies are essential to validate these effects and inform future clinical translation.

**Systematic Review Registration:**

https://www.crd.york.ac.uk/PROSPERO/view/CRD420251002491 identifier CRD420251002491.

## Graphical Abstract

**Figure:**
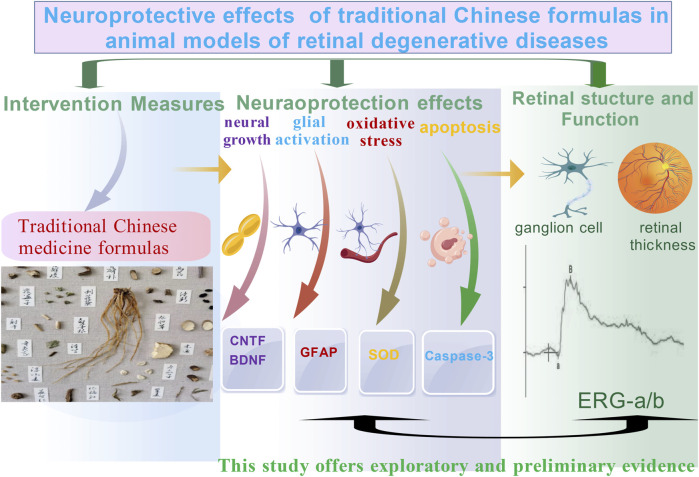
The graphical abstract was created by using BioGDP.com (Jiang et al., 2025), and no copyright issues are involved.

## 1 Introduction

Retinal degenerative diseases (RDDs), a class of progressive blindness disorders such as age-related macular degeneration (AMD), inherited retinal diseases, and retinitis pigmentosa (RP) (De Lima-Vasconcellos et al., 2026), are characterized by the irreversible deterioration of photoreceptors and loss of retinal neurons. Progressive neural degeneration acts as a critical driver of pathogenesis and is mediated by dysregulated neural networks, compromised intercellular communication, redox imbalance, and chronic inflammatory responses in RDDs (Liu B. et al., 2025). Dysfunction of neuroprotection to the retina exacerbates oxidative damage and activates cell death pathways, ultimately leading to retinal neuron injury and functional impairment. Current therapeutic strategies offer limited efficacy and potential for the restoring the vision of individuals with RDDs, underscoring the critical demand for alternative and complementary therapeutic approaches that are capable of halting disease progression and restoring retinal neuroprotection.

Neuroprotection involves the regulation of neuronal growth, glial activation, oxidative stress, and apoptosis and represents a vital therapeutic strategy for preserving retinal integrity (Gage, 2025). Neural growth serves as one of the pivotal molecular regulators of neuroprotection, directly influencing the neurogenic process in the retina by enhancing survival, proliferation, and synaptic plasticity. Key neurotrophic factors such as brain-derived neurotrophic factor (BDNF) and ciliary neurotrophic factor (CNTF) play critical roles in mediating these effects. Glial fibrillary acidic protein (GFAP) is a key activation marker of astrocytes, whose expression levels indicate the physiological or pathological state of retinal glial cells, critically regulating neural circuit function. Oxidative stress is a major pathological driver of retinal disorders and is characterized by an imbalance between reactive oxygen species production and antioxidant defences (Markitantova and Simirskii, 2025). Elevated levels of malondialdehyde, a marker of lipid peroxidation, and altered superoxide dismutase (SOD) activity, a key antioxidant enzyme, reflect an imbalance between ROS production and cellular defences (Hu et al., 2024). This oxidative imbalance induces retinal damage and serves as a fundamental pathological driver of disease progression and plays a significant role in the development, degeneration, and dysfunction of the retinal pigment epithelium. As a tightly regulated form of cell death, apoptosis significantly contributes to retinal dysfunction through selective neuronal loss in retinal layers. Following exposure to blue light, retinal tissues undergo structural and functional apoptosis, characterized by damage to the retinal pigment epithelium, photoreceptors, and ganglion cells, along with increased immunoreactivity of the key apoptotic marker cysteine-dependent aspartate-specific protease 3 (caspase-3) (Ahmed et al., 2024). Therefore, therapeutic strategies aimed at promoting neuroprotection, including modulating glial activation, promoting neuronal growth, and inhibiting oxidative stress and apoptosis, may enhance functional and structural recovery in RDDs.

Traditional Chinese medicine (TCM) formulas have been widely employed in the management of neurodegenerative diseases because of their neuroprotective abilities. Emerging evidence suggests that specific TCM formulas can enhance neuroprotection by attenuating neuronal damage, oxidative stress, and cell apoptosis (Zhu et al., 2022). For instance, the TCM formula Sini Decoction plus Ginseng Soup was reported to shift immune cell metabolism from oxidative phosphorylation to glycolysis, offering a potential therapeutic strategy for retinal degeneration (Zhang et al., 2025). A clinical trial demonstrated that combining the TCM formula Ping-kang granules with methylcobalamin significantly improved neuropathic symptoms in diabetic polyneuropathy patients without severe adverse events (Ga et al., 2025). A randomized controlled trial also suggested that the TCM formula Xiao-Ke-Tong-Bi demonstrated clinical neuroprotective potential in diabetic neuropathy by significantly improving nerve conduction velocities with fewer adverse effects (Lu et al., 2022). Recent clinical studies have also shown that the TCM formula Di-Huang-Yin-Zi have the potential to accelerate nerve repair and could serve as effective adjuvant therapies to enhance the recovery of nerve function in patients with traumatic cauda equina injury (Yu et al., 2025). Accordingly, TCM formulas may exert neuroprotective effects by reducing neuronal damage, improving nerve conduction, and accelerating nerve repair under various neurological conditions. However, few studies have systematically explored the neuroprotective effects of TCM formulas in RDDs.

Therefore, this study adopted an integrated analytical approach combining systematic review, pairwise meta-analysis and network meta-analysis (NMA) to quantitatively assess the treatment efficacy across studies and elucidate potential preferable interventions across studies, contributing to the establishment of a preliminary translational guide for clinical application.

## 2 Methods

### 2.1 Study design

The protocol of this systematic review and meta-analysis was prospectively registered in PROSPERO (ID: CRD420251002491). This systematic review was performed in accordance with the Cocrane Handbook for preferred reporting items for systematic reviews and meta-analyses (PRISMA) statement and the PRISMA for network meta-analyses statement (Page et al., 2021; Hutton et al., 2015). The PRISMA 2020 checklist is provided in the Supplementary Material.

### 2.2 Search strategy

Electronic databases, including the PubMed, Web of Science, Cochrane Library, Embase, China National Knowledge Infrastructure (CNKI), WanFang database, VIP Medical Information, and Chinese Biomedical Literature (CBM) databases, were searched for three conceptual clusters. Studies published in English and Chinese were included from the inception of each database until the final search date. The search strategy used for this review was based on the following terms: “Retinal degenerative diseases,” “BDNF,” “CNTF,” “GFAP,” “SOD,” “Caspase-3,” and “traditional Chinese medicine formula.” Two researchers conducted separate searches and manual retrieval to search for all relevant research literature. The detailed retrieval strategies for several databases are displayed in Supplementary Data Sheet S1.

### 2.3 Eligibility criteria

1. Inclusion criteria: This systematic review and meta-analysis was conducted in accordance with the pre-specified protocol and was structured using the PICOTS framework, which was specifically tailored for synthesising preclinical evidence. All randomized studies meeting the following eligibility criteria were included:
  - Participants: Animal models of RDDs (e.g., RP, AMD, retinal ischaemia‒reperfusion injury, diabetic retinopathy (DR), glaucoma, traumatic optic neuropathy, acute elevated intraocular pressure, retinal vein occlusion and retinal light damage).
  - Interventions: Studies used TCM formulas in experimental group. The key active constituents of the formulas could be identified and validated by TCM systems pharmacology databases, UPLC-Q-TOF-MS/MS, or published studies.
  - Comparisons: Studies used placebo in model control groups.
  - Outcomes: The primary outcomes were neuroprotective indicators, such as “GFAP,” “BDNF,” “CNTF,” “SOD,” and “Caspase-3,” which are expressed as the means ± standard deviations. The secondary outcomes were ophthalmological indicators, including “retinal thickness,” “RGC counts” and “ERG-a/b wave amplitudes,” which were continuous outcomes expressed as the means ± standard deviations.
  - Timeframe: The treatment durations and time points of the outcome assessments were not restricted.
  - Setting: Preclinical investigations were conducted in controlled laboratory settings.
2. Exclusion criteria: Studies meeting any of the following exclusion criteria were excluded:
  - Study types: Studies that did not conform to this research topic, such as s case reports, network pharmacology, reviews, repeated publications, editorials, conference abstracts, or unpublished data.
  - Participants: Studies that did not investigate animal models of RDDs.
  - Intervention: Studies that did not use TCM formulas as the primary intervention (e.g., the use of biomedicines, laser therapy, transcutaneous electrical stimulation, single herbal or botanical extracts as the primary intervention) and lacked documented identification and validation of the key active constituents of the formulas.
  - Outcomes: studies that did not involve measurable outcomes related to neuroprotective effects or lacked corresponding data.

### 2.4 SYRCLE’s tool for assessing risk of bias and quality

The risk of bias in the included studies was assessed by two independent reviewers using the Systematic Review Centre for Laboratory Animal Experiments (SYRCLE) Risk of Bias Tool, which evaluates 10 domains, including selection bias, performance bias, detection bias, attrition bias, reporting bias, and other sources of bias, with each domain rated as “low,” “high,” or “unclear” (Hooijmans et al., 2014).

### 2.5 Procedure for study selection and data extraction

The initial screening of the generated database was conducted by two researchers on the basis of the titles and abstracts. The full texts were subsequently retrieved and independently assessed in detail by two researchers. Information, namely, the first author, year, animal species, age, sex, weight of the animals, groups, number of samples, the methods for establishing RDD models, intervention parameters, continuous data of the outcomes, and course of treatment and adverse reactions, was subsequently independently extracted by two researchers. Any discrepancies during the screening or data extraction process were resolved through discussion or arbitration by a third reviewer.

### 2.6 Strategy for data synthesis and analysis

The meta-analysis was performed by using RevMan 5.4 software. The standardized mean difference (SMD) with 95% confidence intervals was calculated. All the estimates were derived with 95% confidence intervals. Statistical heterogeneity was assessed using the I^2^ statistics. When the heterogeneity was less than 50% (I^2^ < 50%), a fixed-effects model was used; when the heterogeneity exceeded 50% (I^2^ > 50%), a random-effects model was used.

### 2.7 Sensitivity and subgroup analyses to explore possible causes of heterogeneity

To explore potential sources of heterogeneity, sensitivity analysis was performed using STATASE 16 software by sequentially excluding each individual study to assess the stability and robustness of the pooled results regarding the neuroprotective effects of TCM formulas in animal models of RDDs. To further explore potential sources of heterogeneity, subgroups were defined on the basis of animal species, disease models, types of intervention, dosage levels, and durations of treatment, among other factors.

### 2.8 Publication bias

The value of Pr > |t| in Begg’s test and Pr > |t| in Egger’s test were employed to assess the publication bias in the included outcomes. A significance level of p < 0.05 in either Begg’s test or Egger’s test was considered indicative of statistically significant publication bias. Trim and Fill method in STATASE was planned to further assess and adjust for potential publication bias.

### 2.9 Bayesian NMA analysis process

This NMA was conducted as an extension analysis of pairwise meta-analysis in this study. This NMA analysis was conducted in accordance with the pre-specified protocol and was structured using the PICOTS framework. The detailed PICOTS criteria are as follows:

- Participants: Animal models of RDDs.
- Interventions: Studies used TCM formulas, especially with high dose formulas in experimental group.
- Comparisons: Studies used either placebo, TCM formulas distinct from the experimental group, low-dose TCM formulas in experimental group, middle-dose TCM formulas in experimental group, or biomedicines in control groups.
- Outcomes: The outcomes include “GFAP”, “BDNF”, “CNTF,” “SOD,” “Caspase-3,” “apoptotic cell counts,” “retinal thickness,” “RGC cell counts” and “ERG-a/b wave amplitudes,” which were continuous outcomes expressed as the means ± standard deviations.
- Timeframe: The treatment durations and time points of the outcome assessments were not restricted.
- Setting: Preclinical investigations were conducted in controlled laboratory settings.

In this NMA analysis, random-effect model was employed for effect size pooling using the multinma package in R 4.5.1, with statistical significance set at P < 0.05. Network plots of treatment interventions were constructed with the model group serving as the common reference node. The transitivity assumption was pre-specified in our study. This assumption requires that included studies demonstrate sufficient methodological similarity to permit valid comparison of intervention effects across the network. To evaluate the transitivity of the included studies, the distribution of potential effect modifiers (e.g., animal species, RDD models, focus areas) across treatment comparisons would be systematically assessed. A summary table would be provided to illustrate the distribution of modifiers across all comparisons. The transitivity assumption would be considered supported if no severe systematic imbalances in key modifiers were observed. The consistency between direct and indirect evidence was evaluated globally by comparing the fit of consistency and inconsistency (unrelated mean effects, UME) models using the deviance information criterion (DIC). A ΔDIC of less than 5 was considered to support the consistency model. Any deviations from these pre-specified assessment methods would be explicitly mentioned in the results.

### 2.10 Characterization and validation of TCM formulas

The herbal compositions of the included TCM formulas were extracted from the original studies. All species must be validated taxonomically in the TCM formulas in Medicinal Plant Names Services (MPNS) to verify the full species names, authorities and families. All TCM formulas were evaluated for the quality of their composition reporting using the ConPhYMP tool. The ConPhYMP checklists are provided in Supplementary Material (ConPhyMP-checklists-table-1 and ConPhyMP-checklists-table-2a).

## 3 Results

### 3.1 Literature search

**FIGURE 1:**
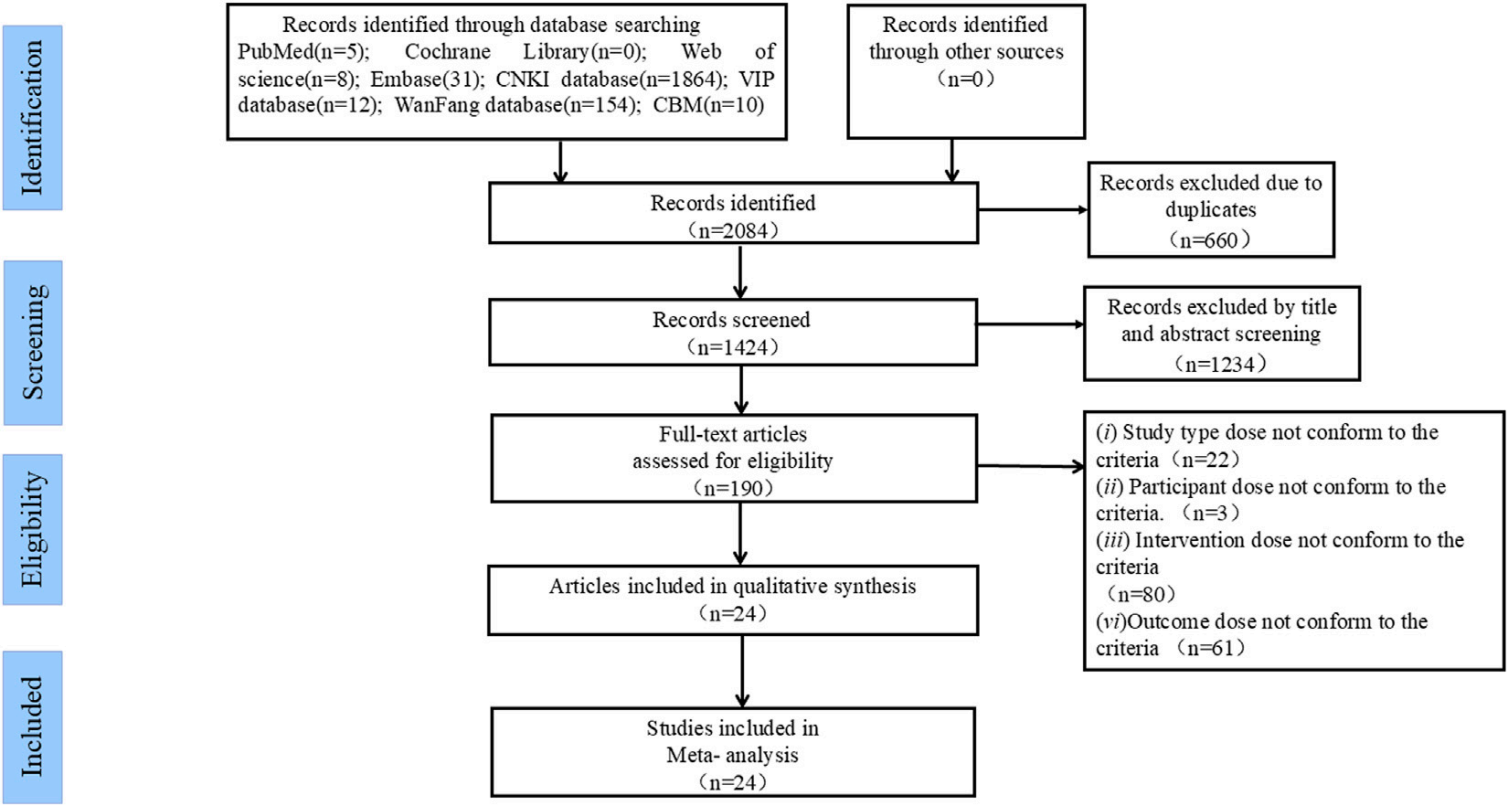
The inclusion and exclusion process.

By screening 8 electronic databases, 2084 studies that were potentially eligible for this study were initially obtained. After duplicate articles were removed, 660 studies remained. Among the 1424 studies, 1234 irrelevant studies were excluded after their titles and abstracts were reviewed. Afterwards, the remaining 190 studies were assessed according to the inclusion and exclusion criteria by carefully reading the full text. Ultimately, 24 studies were confirmed to be eligible for inclusion in this meta-analysis (Hou, 2017; Xu et al., 2021; Chen et al., 2006; Chen, 2021; Sun, 2010; Zhang, 2009; Yao et al., 2012; Liu et al., 2024; Ye et al., 2004; Fang, 2020; Shi et al., 2022a; Shi et al., 2022b; Miao, 2023; Wu et al., 2019; Cui, 2009; Xu et al., 2016; Deng, 2010; Qin, 2018; Ma, 2005; Yang, 2003; Bao et al., 2018; Che, 2020; Ding, 2022; Wang et al., 2011). The inclusion and exclusion process for each selected study is shown in Figure 1.

### 3.2 Main characteristics of the studies and interventions

The detailed characteristics of the 24 included studies are summarized in Table 1. The included studies focused mainly on Sprague‒Dawley (SD) rats and C57BL/6 mouse models. Retinal diseases range from retinal vascular and cellular injury (e.g., AMD, retinal vein occlusion, DR, and retinal ischaemia‒reperfusion injury) to optic nerve injuries (e.g., glaucoma, optic nerve injury, and acute elevated intraocular pressure). The methods for establishing RDDs range from mechanical injury models (e.g., traumatic optic neuropathy) to non-mechanical injury models (e.g., retinal light damage). The treatment durations vary from short-term to long-term regimens. Key evaluation indicators include BDNF, CNTF, GFAP, SOD, caspase-3, retinal apoptotic cell counts, ERG-a wave, ERG-b wave, RGC counts, and retinal thickness.

**TABLE 1:** The detailed characteristics of the 24 included studies.

No.	Included studies	Animal	Diseases	Number of experimental animals (n)	Intervention measures				Outcomes	Course of	Focus area
					Experimental group	Positive control group 1	Positive control group 2	Positive control group 3		Treatment	
1	Liu et al. (2024)	Db/db mice	Diabetic retinopathy	36	Bu-Shen-Huo-Xue Decoction	Qi-Ming Granule	—	—	GFAP, retinal apoptotic cells	8 weeks	Retina
2	Miao (2023)	C57BL/6J mice	Retinal vein occlusion	96	High dose Qu-Ji-Tong-Luo Decoction	Middle dose Qu-Ji-Tong-Luo Decoction	Low dose Qu-Ji-Tong-Luo Decoction	—	GFAP	2 weeks	Retina
3	Ding (2022)	SD rat	Retinal vein occlusion	30	High dose Qu-Yu-Tong-Luo Prescription	Middle dose Qu-Yu-Tong-Luo Prescription	Low dose Qu-Yu-Tong-Luo Prescription	—	RGC counts, GFAP, retinal thickness, retinal apoptotic cells, ERG a/b wave	2 weeks	Retina
4	Shi et al. (2022b)	SD rat	Glaucoma optic neuropathy	60	Qing-Guang-An Granule	—	—	—	Retinal apoptotic cells	3 months	Optic nerve, retina
5	Shi et al. (2022a)	DBA/2J	Glaucoma optic neuropathy	48	Qing-Guang-An-Granule-Ⅱ	Yi-Mai-Kang Dispersible Tablets	—	—	Caspase-3	4 weeks	Optic nerve, retina
6	Chen (2021)	SD rat	Retinal light damage	25	High dose Bu-Yang-Huan-Wu Decoction	Middle dose Bu-Yang-Huan-Wu Decoction	Low dose Bu-Yang-Huan-Wu Decoction	—	RGC counts, retinal thickness, ERG a/b wave	3 weeks	Retina
7	Xu et al. (2021)	C57BL/6 mice	Dry age-related macular degeneration	36	Bu-Shen-Yi-Jing-Fang	—	—	—	CNTF	2 weeks	Retina
8	Fang (2020)	SD rat	Diabetic retinopathy	35	Ming-Mu-Xiao-Meng tablet	Calcium Dobesilate Capsules	—	—	ERG a/b wave	3 months	Retina
9	Che (2020)	SD rat	Glaucoma optic neuropathy	30	High dose Zi-Yin-Ming-Mu Decoction	Middle dose Zi-Yin-Ming-Mu Decoction	Low dose Zi-Yin-Ming-Mu Decoction	Qi-Ju-Di-Huang Pills	GFAP, RGC counts, retinal thickness, retinal apoptotic cells	4 weeks	Optic nerve, retina
10	Wu et al. (2019)	SD rat	diabetic retinopathy	120	Mi-Meng-Hua-Compound Prescription	—	—	—	ERG a/b wave, BDNF, CNTF	90 days	Retina
11	Bao et al. (2018)	SD rat	Retinal ischemia-reperfusion injury	40	Zhen-Bao-Wan	Procyanidins	—	—	BDNF	1 week	Retina
12	Qin (2018)	SD rat	Retinal ischemia-reperfusion injury	40	Yi-Qi-Wen-Yang-Tong-Luo Decoction	Alprostadil	—	—	Retinal thickness, SOD,ERG a/b wave	4 weeks	Retina
13	Hou (2017)	C57BL/6 mice	Dry age-related macular degeneration	72	Bu-Shen-Yi-Jing-Fang	—	—	—	ERG a/b wave, CNTF	4 weeks	Retina
14	Xu et al. (2016)	SD rat	Traumatic optic neuropathy	208	Xue-Fu-Zhu-Yu Decoction	Neurotrophic factor	Xue-Fu-Zhu-Yu Decoction and Neurotrophic factor	—	RGC counts	28 days	Optic nerve, retina
15	Yao et al. (2012)	SD rat	Diabetic retinopathy	8	High dose Huo-Xue-Jie-Du Recipe	Middle dose Huo-Xue-Jie-Du Recipe	Low dose Huo-Xue-Jie-Du Recipe	Calcium Dobesilate Capsules	GFAP,ERG a/b wave	20 weeks	Retina
16	Wang et al. (2011)	SD rat	Traumatic optic neuropathy	40	Zuo-Gui Pill	—	—	—	GFAP, RGC counts	16 weeks	Optic nerve, retina
17	Sun (2010)	SD rat	Acute elevated intraocular pressure	90	Huo-Xue-Hua-Yu Decoction	—	—	—	BDNF	2 weeks	optic nerve
18	Deng (2010)	SD rat	Diabetic retinopathy	20	Qi-Shen-Yi-Qi Pills	—	—	—	GFAP	300 days	Retina
19	Cui (2009)	SD rat	Traumatic optic neuropathy	60	Xue-Fu-Zhu-Yu Decoction	Erigeron breviscapus aqueous solution	—	—	Retinal thickness	4 weeks	Optic nerve, retina
20	Zhang (2009)	Wister rat	Acute elevated intraocular pressure	100	Huo-Xue-Hua-Yu Decoction	—	—	—	BDNF	2 weeks	Optic nerve, retina
21	Chen et al. (2006)	SD rat	Retinal ischemia-reperfusion injury	30	Bu-Yang-Huan-Wu Decoction	—	—	—	Retinal thickness	10 days	Retina
22	Ma (2005)	SD rat	Retinal light damage	40	High dose Yishi-Tablet	Low dose Yishi-Tablet	—	—	Caspase-3	5 weeks	Retina
23	Ye et al. (2004)	SD rat	Retinal light damage	32	High dose Ming-Mu-Wu-Zi	Middle dose Ming-Mu-Wu-Zi	—	—	Retinal apoptotic cells	2 weeks	Retina
24	Yang (2003)	SD rat	Retinal light damage	50	High dose Yishi-Tablet	Middle dose Yishi-Tablet	Low dose Yishi-Tablet	—	SOD,ERG a/b wave	4 weeks	Retina

Detailed information on the included TCM formulas, including the representative herbal compositions by Chinese name, botanical names of composition by MPNS validation, representative bioactive compounds linked to the therapeutic effects, production or procurement sources, and pharmacological effects, is detailed in Table 2. Table 2 defines the included TCM formulas and provides a transparent, scientifically-grounded basis for the formulas under investigation. For instance, Bu-Shen-Huo-Xue decoction (Liu et al., 2024) is composed of *DiHuang*, *DanShen*, *GeGen*, *RenShen* (*Rehmannia glutinosa* Libosch. [Scrophulariaceae, Rehmanniae radix],*Salvia miltiorrhiza* Bunge [Lamiaceae, Salviae miltiorrhizae radix et rhizoma], *Pueraria lobata* (Willd.) Ohwi [Fabaceae, Puerariae lobatae radix], *Panax ginseng C.A*. Meyer [Araliaceae, Ginseng radix et rhizoma]). The representative bioactive compounds of the Bu-Shen-Huo-Xue decoction are verbascoside, cryptotanshinone, puerarin, and ginsenosides Re,Rd and Rb1, which exhibits anti-apoptotic and glial activation-reducing effects in RDD models.

**TABLE 2:** The composition and validation of the TCM formulas.

No.	Included studies	Included TCM formulas	Representative herbal compositions in included formulas by Chinese name	Botanical names of composition by MPNS validation	Representative validated bioactive compounds linked to the therapeutic effects	Pharmacological and therapeutic effects	Production or procurement sources
1	Liu et al. (2024)	Bu-Shen-Huo-Xue Decoction	*DiHuang*, *DanShen*, *GeGen*, *RenShen*	*Rehmannia glutinosa* Libosch. [Scrophulariaceae, Rehmanniae radix]*,Salvia miltiorrhiza* Bunge [Lamiaceae, Salviae miltiorrhizae radix et rhizoma]*,Pueraria lobata* (Willd.) Ohwi [Fabaceae, Puerariae lobatae radix]*,Panax ginseng C.A*.Meyer [Araliaceae, Ginseng radix et rhizoma]	Verbascoside, Cryptotanshinone, Puerarin, Ginsenoside Re, Rd and Rb1	Exhibits anti-apoptotic and glial activation-reducing effects in RDD model	Chengdu Lotus Pond TCM Market, authenticated by Prof. Pei Jin (CDUTCM)
2	Miao (2023)	Qu-Ji-Tong-Luo Decoction	*TaoRen*, *HongHua*, *DiHuang*, *DangGui*, *JiNeiJing*, *FaBanXia*, *ChenPi*, *FuLing*, *SanQ*i, *FangFeng*	*Prunus persica (L.)* Batsch [Rosaceae, Persicae semen]*,Carthamus tinctorius* L. [Asteraceae, Carthami flos]*,Rehmannia glutinosa* Libosch. [Scrophulariaceae, Rehmanniae radix]*,Angelica sinensis* (Oliv.) Diels [Apiaceae, Angelicae sinensis radix]*,Gallus domesticus* Brisson [Phasianidae, Endothelium corneum gigeriae galli]*,Pinellia ternata* (Thunb.) Makino [Araceae, Pinelliae tuber]*,Citrus reticulata* Blanco [Rutaceae, Citri reticulatae pericarpium]*,Poria cocos* (Schwein.) F.A. Wolf [Polyporaceae, Poriae sclerotium]*,Panax notoginseng* (Burkill) F.H. Chen ex C.Y. Wu et K.M. Feng [Araliaceae, Notoginseng radix et rhizoma (pulvis)]*,Saposhnikovia divaricata* (Turcz.) Schischk. [Apiaceae, Saposhnikoviae radix]	Hesperetin, keratin, Baicalein, Naingenin, Wogonin	Exhibits glial activation-reducing effects in RDD model	China Academy of Chinese Medical Sciences Ophthalmic Hospital Pharmacy Department
3	Ding (2022)	Qu-Yu-Tong -Luo Prescription	*PuHuang*, *BaiZhi*, *GeGen*, *JiangHuang*, *TaoRen*, *WuLingZhi, HongHua*, *DaHuang*, *ChuanXiong*, *ShuiZhi*	*Typha angustifolia* L. [Typhaceae; Typhae pollen], *Angelica dahurica* (Fisch. ex Hoffm.) Benth. and Hook.f. [Apiaceae; Angelicae dahuricae radix]*, Pueraria montana* var. thomsonii (Benth.) M.R.Almeida [Fabaceae; Puerariae lobatae radix]*, Curcuma longa* L. [Zingiberaceae; Curcumae longae rhizoma], *Prunus persica* (L.) Batsch [Rosaceae; Persicae semen], *Trogopterus xanthipes* Milne-Edwards [Petauristidae; Trogopterori faeces]*,Carthamus tinctorius* L. [Asteraceae; Carthami flos]*,Rheum palmatum *L. [Polygonaceae; Rhei radix et rhizoma], *Ligusticum chuanxiong* Hort. [Apiaceae; Chuanxiong rhizoma],*Hirudo nipponica* Whitman [Hirudinidae; Hirudo]	Quercetin, Kaempferol,Byakangelicol, Isoimperatorin,formononetin,β-sitosterol,Stigmasterol, campesterol,Baicalein, 6-Hydroxykaempferol, Flavoxanthin, Sitosterol	Exhibits anti-apoptotic and glial activation-reducing effects, protects retinal functions and structures in RDD model	The Second Affiliated Hospital of Liaoning University of Traditional Chinese Medicine
4	Shi et al. (2022b)	Qing-Guang-An Granule	*HuangQ*i, *FuLing*, *BaiZhu*, *ChiShao*, *DiHuang*, *DiLong*, *HongHua*, *CheQianZi*	*Astragalus membranaceus* (Fisch.) Bge. [Fabaceae, Astragali radix], *Poria cocos* (Schwein.) F.A. Wolf [Polyporaceae, Poriae sclerotium], *Atractylodes macrocephala* Koidz. [Asteraceae, Atractylodis macrocephalae rhizoma],*Paeonia veitchii* Lynch [Ranunculaceae, Paeoniae veitchii radix],*Rehmannia glutinosa* Libosch. [Scrophulariaceae, Rehmanniae radix],*Pheretima aspergillum* (E. Perrier) [Megascolecidae, Pheretima],*Carthamus tinctorius* L. [Asteraceae, Carthami flos],*Plantago asiatica* L. [Plantaginaceae, Plantaginis asiaticae herba]	Quercetin, Kaempferol, Luteolin, 7-O-methylisomucronulatol, Baicalein, β-sitosterol, Stigmasterol, Formononetin, Isorhamnetin	Exhibits anti-apoptotic effects in RDD model	Hunan University of Chinese Medicine First Affiliated Hospital
5	Shi et al. (2022a)	Qing-Guang-An Granule-Ⅱ	*GouQiZi*, *NiuXi*, *NvZhenZi* *DengZhanXiXin*, *HuangQi*	*Lycium chinense* Mill. [Solanaceae, Lycii fructus],*Achyranthes bidentata* Blume [Amaranthaceae, Achyranthis bidentatae radix],*Ligustrum lucidum* Ait. [Oleaceae, Ligustri lucidi fructus],*Erigeron breviscapus* (Vaniot) Hand.-Mazz. [Asteraceae], *Astragalus membranaceus* (Fisch.) Bge. [Fabaceae, Astragali radix]	Lycium barbarum polysaccharides, Formononetin Quercetin, Kaempferol, Baicalein, Luteolin	Exhibits anti-apoptotic effects in RDD model	Not mentioned
6	Chen (2021)	Bu-Yang-Huan-Wu Decoction	*HuangQi*, *DangGui*, *ChiShao*, *DiLong*, *ChuanXiong*, *TaoRen*, *HongHua*	*Astragalus membranaceus* (Fisch.) Bge. [Fabaceae, Astragali radix], *Angelica sinensis* (Oliv.) Diels [Apiaceae, Angelicae sinensis radix], *Paeonia veitchii* Lynch [Ranunculaceae, Paeoniae radix rubra], *Pheretima aspergillum* (E. Perrier) [Megascolecidae, Pheretima], *Ligusticum chuanxiong* Hort. [Apiaceae, Chuanxiong rhizoma], *Prunus persica* (L.) Batsch [Rosaceae, Persicae semen]*, Carthamus tinctorius* L. [Asteraceae, Carthami flos]	Calycosin, Quercetin, Kaempferol, β-sitosterol and Stigmasterol	Protects retinal functions and structures	Sichuan New Green Pharmaceutical Technology Development Co., Ltd
7	Xu et al. (2021)	Bu-Shen-Yi -Jing-Fang	*HeShouWu*, *GouQi*, *HuangQi*, *HuangJing*	*Reynoutria multiflora* (Thunb.) Hara [Polygonaceae, Polygoni multiflori radix]*,Lycium chinense* Mill. [Solanaceae, Lycii fructus]*,Astragalus membranaceus* (Fisch.) Bge. [Fabaceae, Astragali radix]*,Polygonatum odoratum* (Mill.) Druce [Asparagaceae, Polygonati rhizoma]	Lycium barbarum polysaccharides, quercetin, kaempferol,isoflavanone, formononetin,Baicalein, Diosgenin, Methylprotodioscin_qt	Enhances neural growth in RDD models	Not mentioned
8	Fang (2020)	Ming-Mu-Xiao-Meng Tablet	*RenShen, MaiDong, WuWeiZi, BanXia, ChenPi, FuLíng, GanCao, ZhuRu, ZhiShi, ShengJiang, DaZao, JiLi, MiMengHua, MaoDongQing, WaLengZi*	*Panax ginseng* C.A. Meyer [Araliaceae, Ginseng radix et rhizoma]*,Ophiopogon japonicus* (Thunb.) Ker-Gawl. [Asparagaceae, Ophiopogonis japonicus radix]*,Schisandra chinensis* (Turcz.) Baill. [Schisandraceae, Schisandrae chinensis fructus]*,Pinellia ternata* (Thunb.) Makino [Araceae, Pinelliae tuber]*, Citrus reticulata* Blanco [Rutaceae, Citri reticulatae pericarpium]*,Poria cocos* (Schwein.) F.A. Wolf [Polyporaceae, Poriae sclerotium]*,Glycyrrhiza uralensis* Fisch. [Fabaceae, Glycyrrhizae radix et rhizoma]*,Bambusa textilis* McClure [Poaceae, Bambusae caulis in taeniam]*,Citrus aurantium* L. [Rutaceae, Aurantii fructus immaturus]*,Zingiber officinale* Roscoe [Zingiberaceae, Zingiberis rhizoma recens]*,Ziziphus jujuba* Mill. [Rhamnaceae, Jujubae fructus]*,Tribulus terrestris* L. [Zygophyllaceae, Tribuli fructus]*,Buddleja officinalis* Maxim. [Scrophulariaceae, Buddlejae flos]*,Ilex pubescens* Hook. et Arn. [Aquifoliaceae, Ilicis pubescentis radix]*,Arca inflata* Reeve [Arcidae, Arcae concha]	Ginsenoside Rb1,Betaine, Hesperidin,Notoginsenoside R1	Protects retinal functions in RDD model	Department of Pharmacy, Eye Hospital, China Academy of Chinese Medical Sciences
9	Che (2020)	Zi-Yin-Ming-Mu Decoction	*DiHuang*, *GouQiZi*, *GeGen*, *DangGui*, *ChuanXiong*, *TuSiZi*, *CheQianZi*, *WuWeiZi*, *ChuShiZi*, *GanCao*	*Rehmannia glutinosa* Libosch. [Scrophulariaceae, Rehmanniae radix praeparata],*Lycium chinense* Mill. [Solanaceae, Lycii fructus],*Pueraria lobata* (Willd.) Ohwi [Fabaceae, Puerariae lobatae radix],*Angelica sinensis* (Oliv.) Diels [Apiaceae, Angelicae sinensis radix],*Ligusticum chuanxiong* Hort. [Apiaceae, Chuanxiong rhizoma],*Cuscuta chinensis* Lam. [Convolvulaceae, Cuscutae semen],*Plantago asiatica* L. [Plantaginaceae, Plantaginis semen],*Schisandra chinensis* (Turcz.) Baill. [Schisandraceae, Schisandrae chinensis fructus],*Broussonetia papyrifera* (L.) L'Hér. ex Vent. [Moraceae, Broussonetiae fructus],*Glycyrrhiza uralensis* Fisch. [Fabaceae, Glycyrrhizae radix et rhizoma]	Sitosterol, Stigmasterol Cycloartenol, Ethyl linolenate, Mandenol,Cyanin Formononetin, Kaempferol, Isorhamnetin,Sesamin, (11E,13E)-Docosa-11,13-dienoic acid	Exhibits anti-apoptotic and glial activation-reducing effects, protects retinal structures in RDD model	Shenyang He’s Eye Hospital Chinese Medicine Pharmacy
10	Wu et al. (2019)	Mi-Meng-Hua-Compound Prescription	*HuangQi*, *NvZhenZi*, *YiMuCao*, *HuangLian*, *RouGui*, *MiMengHua*	*Astragalus membranaceus* (Fisch.) Bge. [Fabaceae, Astragali radix], *Ligustrum lucidum* Ait. [Oleaceae, Ligustri lucidi fructus], *Leonurus japonicus* Houtt. [Lamiaceae, Leonuri herba], *Coptis chinensis* Franch. [Ranunculaceae, Coptidis rhizoma],*Cinnamomum cassia* Presl [Lauraceae, Cinnamomi cortex], *Buddleja officinalis* Maxim. [Scrophulariaceae, Buddlejae flos]	Linarin, Verbascoside, Luteolin Salidroside, Isoverbascoside	Enhances neural growth, protects retinal functions in RDD model	Beijing Yanbei Medicinal Materials Company (Batch No.: 121016)
11	Bao et al. (2018)	Zhen-Bao-Wan	*ZiTan*, *ZhenZhu*, *NiuHuang*, *XiJiao*, *SheXiang, JiangXiang, HongHua*	*Pterocarpus santalinus* L. [Fabaceae, Pterocarpi santalini lignum],*Pinctada martensii* (Dunker) [Pteriidae, Margarita],*Bos taurus domesticus* Gmelin [Bovidae, Calculus bovis]*,Rhinoceros unicornis* L. [Rhinocerotidae, Cornu rhinocerotis],*Moschus berezovskii* Flerov [Moschidae, Moschus]*,Dalbergia odorifera* T.C.Chen [Fabaceae],*Carthamus tinctorius* L. [Asteraceae, Carthami flos]	β-sitosterol, Sitosterol, Stigmasterol, Liquiritigenin, Cryptochlorogenic acid, Ganoderic acid DM, Isochlorogenic acid A	Enhances neural growth in RDD model	National Mongolian Medicinal Preparation Center of Inner Mongolia International Mongolian Medicine Hospital (Approval No.: NM ZMZ M1401081)
12	Qin (2018)	Yi-Qi-Wen -Yang-Tong-Luo Decoction	*Huangqi*, *DangGui*, *HongJingTian*, *GuiZhi*, *ChuanXiong*, *DiLong*	*Astragalus membranaceus* (Fisch.) Bge. [Fabaceae, Astragali radix],*Angelica sinensis* (Oliv.) Diels [Apiaceae, Angelicae sinensis radix],*Rhodiola crenulata* (Hook. f. et Thoms.) H. Ohba [Crassulaceae, Rhodiolae crenulatae radix et rhizoma]*,Cinnamomum cassia P*resl [Lauraceae, Cinnamomi ramulus]*,Ligusticum chuanxiong* Hort. [Apiaceae, Chuanxiong rhizoma],*Pheretima aspergillum* (E. Perrier) [Megascolecidae, Pheretima]	Isoflavanone, β-sitosterol, Stigmasterol, Taxifolin, Mandenol, Sitosterol	Exhibits antioxidant stress effects, protects retinal functions and structures in RDD model	China-Japan Friendship Hospital Traditional Chinese Medicine Decoction Room
13	Hou (2017)	Bu-Shen-Yi-Jing- Fang	*HeShouWu*, *GouQi,Zi*, *HuangJing*	*Reynoutria multiflora* (Thunb.) Hara [Polygonaceae, Polygoni multiflori radix]*,Lycium barbarum* L. [Solanaceae, Lycii fructus],*Polygonatum sibiricum* Redouté [Asparagaceae, Polygonati sibirici rhizoma]	Quercetin, Luteolin,Kaempferol, Isorhamnetin	Enhances neural growth, protects retinal functions in RDD model	Chinese Medicine Pharmacy, Ophthalmic Hospital, China Academy of Chinese Medical Sciences
14	Xu et al. (2016)	Xue-Fu-Zhu-Yu Decoction	*TaoRen*, *HongHua*, *DangGui*, *DiHuang*, *ChuanXiong*, *ChiShao*, *NiuX*i, *JieGeng*, *ChaiHu*, *ZhiQiao*, *GanCao*	*Prunus persica* (L.) Batsch [Rosaceae, Persicae semen],*Carthamus tinctorius* L. [Asteraceae, Carthami flos],*Angelica sinensis* (Oliv.) Diels [Apiaceae, Angelicae sinensis radix],*Rehmannia glutinosa* Libosch. [Scrophulariaceae, Rehmanniae radix],*Ligusticum chuanxiong* Hort. [Apiaceae, Chuanxiong rhizoma],*Paeonia veitchii* Lynch [Ranunculaceae, Paeoniae veitchii radix],*Achyranthes bidentata* Blume [Amaranthaceae, Achyranthis bidentatae radix],*Platycodon grandiflorus* (Jacq.) A. DC. [Campanulaceae, Platycodonis radix],*Bupleurum chinense* DC. [Apiaceae, Bupleuri radix],*Citrus aurantium* L. [Rutaceae, Aurantii fructus immaturus],*Glycyrrhiza uralensis* Fisch. [Fabaceae, Glycyrrhizae radix et rhizoma]	Quercetin, Luteolin, Kaempferol, Wogonin and Naringenin	Protects retinal structures in RDD model	Guiyang Jirentang Pharmaceutical Co., Ltd., Guizhou Province
15	Yao et al. (2012)	Huo-Xue-Jie -Du Recipe	*SanQi, HuangLian* *TianHuaFen* *GuiJianYu*	*Panax notoginseng* (Burkill) F.H. Chen ex C.Y. Wu et K.M. Feng [Araliaceae, Notoginseng radix et rhizoma],*Coptis chinensis* Franch. [Ranunculaceae, Coptidis rhizoma],*Trichosanthes kirilowii* Maxim. [Cucurbitaceae], *Euonymus alatus* (Thunb.) Siebold [Celastraceae]	Notoginsenosides, Ginsenosides, Hyperin, dehydrodicatechin A, Dihydroisocucurbitacin B	Exhibits glial activation-reducing effects, protects retinal functions in RDD model	School of Pharmaceutical Sciences, Beijing University of Chinese Medicine
16	Wang et al. (2011)	Zuo-Gui Pill	*DiHuang*, *ShanYao*, *ShanZhuYu*, *GouQiZi*, *GuiBan*, *NiuXi*, *TuSiZi*, *LuJiaoJiao*	*Rehmannia glutinosa* Libosch. [Scrophulariaceae, *Rehmanniae radix praeparata*],*Dioscorea opposita* Thunb. [Dioscoreaceae, Dioscoreae rhizoma],*Cornus officinalis* Siebold et Zucc. [Cornaceae, Corni fructus],*Lycium chinense* Mill. [Solanaceae, Lycii fructus],*Chinemys reevesii* (Gray) [Testudinidae, Testudinis carapax et plastrum],*Achyranthes bidentata* Blume [Amaranthaceae, Achyranthis bidentatae radix],*Cuscuta chinensis* Lam. [Convolvulaceae, Cuscutae semen],*Cervus nippon* Temminck [Cervidae, Cervi cornus colla]	Quercetin, Kaempferol, β-sitosterol, Isorhamnetin, Stigmasterol	Exhibits glial activation-reducing effects, protects retinal structures in RDD model	Shanghai Yanghetang Traditional Chinese Medicine Decoction Pieces Co., Ltd
17	Sun (2010)	Huo-Xue-Hua-Yu Decoction	*DiHuang, ChiShao, DangGui, ChuanXiong, TaoRen, HongHua, ZhiZi, DanShen, ZeLan, ChongWeiZi, DanPi, GanCao*	*Rehmannia glutinosa* Libosch. [Scrophulariaceae, Rehmanniae radix],*Paeonia veitchii* Lynch [Ranunculaceae, Paeoniae radix rubra], *Angelica sinensis* (Oliv.) Diels [Apiaceae, Angelicae sinensis radix],*Ligusticum chuanxiong* Hort. [Apiaceae, Chuanxiong rhizoma],*Prunus persica* (L.) Batsch [Rosaceae, Persicae semen],*Carthamus tinctorius* L. [Asteraceae, Carthami flos],*Gardenia jasminoides* Ellis [Rubiaceae, Gardeniae fructus],*Salvia miltiorrhiza* Bunge [Lamiaceae, Salviae miltiorrhizae radix et rhizoma],*Lycopus lucidus* Turcz. [Lamiaceae, Lycopi herba],*Leonurus japonicus* Houtt. [Lamiaceae, Leonuri fructus],*Paeonia suffruticosa* Andr. [Ranunculaceae, Moutan cortex],*Glycyrrhiza uralensis* Fisch. [Fabaceae, Glycyrrhizae radix et rhizoma]	Sitosterol, Stigmasterol, Ellagic acid, Paeoniflorin, Baicalein, β-Sitosterol,Mandenol, Lignan, Crocetin, Quercetin, Kaempferol	Enhances neural growth in RDD model	Not mentioned
18	Deng (2010)	Qi-Shen-Yi-Qi Pills	*HuangQi*, *DanShen*, *SanQi*, *JiangXiang*	*Astragalus membranaceus* (Fisch.) Bge. [Fabaceae, Astragali radix],*Salvia miltiorrhiza* Bunge [Lamiaceae, Salviae miltiorrhizae radix et rhizoma],*Panax notoginseng* (Burkill) F.H. Chen ex C.Y. Wu et K.M. Feng [Araliaceae, Notoginseng radix et rhizoma],*Dalbergia odorifera* T.C. Chen [Fabaceae, Dalbergiae odoriferae lignum]	Quercetin, Astragaloside IV, Danshensu, Apigenin, Cryptotanshinone, Luteolin, and Tanshinone IIB.	Exhibits glial activation-reducing effects in RDD model	Tianjin Tianshili Pharmaceutical Co., Ltd
19	Cui (2009)	Xue-Fu-Zhu-Yu Decoction	*TaoRen*, *HongHua*, *DangGui*, *DiHuang*, *ZhiQiao*, *Chishao*, *ChuanXiong*, *ChaiHu*, *JieGeng*, *NiuXi*, *GanCao*	*Prunus persica* (L.) Batsch [Rosaceae, Persicae semen],*Carthamus tinctorius* L. [Asteraceae, Carthami flos].*Angelica sinensis* (Oliv.) Diels [Apiaceae, Angelicae sinensis radix],*Rehmannia glutinosa* Libosch. [Scrophulariaceae, Rehmanniae radix],*Ligusticum chuanxiong* Hort. [Apiaceae, Chuanxiong rhizoma],*Paeonia veitchii* Lynch [Ranunculaceae, Paeoniae veitchii radix],*Achyranthes bidentata* Blume [Amaranthaceae, Achyranthis bidentatae radix],*Platycodon grandiflorus* (Jacq.) A. DC. [Campanulaceae, Platycodonis radix],*Bupleurum chinense* DC. [Apiaceae, Bupleuri radix],*Citrus aurantium* L. [Rutaceae, Aurantii fructus immaturus],*Glycyrrhiza uralensis* Fisch. [Fabaceae, Glycyrrhizae radix et rhizoma]	Quercetin, Luteolin, Kaempferol, Wogonin and Naringenin	Protects retinal structures in RDD model	Not mentioned
20	Zhang (2009)	Huo-Xue-Hua-Yu Decoction	*DiHuang*, *ChiShao*, *DangGui*, *ChuanXiong*, *TaoRen*, *HongHua*, *ZhiZi*, *DanShen*, *ZeLan*, *ChongWeiZi*, *DanPi*, *GanCao*	*Rehmannia glutinosa* Libosch. [Scrophulariaceae, Rehmanniae radix],*Paeonia veitchii* Lynch [Ranunculaceae, Paeoniae radix rubra], *Angelica sinensis* (Oliv.) Diels [Apiaceae, Angelicae sinensis radix],*Ligusticum chuanxiong* Hort. [Apiaceae, Chuanxiong rhizoma],*Prunus persica* (L.) Batsch [Rosaceae, Persicae semen],*Carthamus tinctorius* L. [Asteraceae, Carthami flos],*Gardenia jasminoides* Ellis [Rubiaceae, Gardeniae fructus],*Salvia miltiorrhiza* Bunge [Lamiaceae, Salviae miltiorrhizae radix et rhizoma],*Lycopus lucidus* Turcz. [Lamiaceae, Lycopi herba],*Leonurus japonicus* Houtt. [Lamiaceae, Leonuri fructus],*Paeonia suffruticosa* Andr. [Ranunculaceae, Moutan cortex],*Glycyrrhiza uralensis* Fisch. [Fabaceae, Glycyrrhizae radix et rhizoma]	Sitosterol, Stigmasterol, Ellagic acid, Paeoniflorin, Baicalein, β-Sitosterol, Mandenol, Lignan, Crocetin, Quercetin, Kaempferol	Enhances neural growth in RDD model	Not mentioned
21	Chen et al. (2006)	Bu-Yang-Huan-Wu Decoction	*HuangQi*, *DangGui*, *ChiShao*, *DiLong*, *ChuanXiong*, *TaoRen*, *HongHua*	*Astragalus membranaceus* (Fisch.) Bge. [Fabaceae, Astragali radix],*Angelica sinensis* (Oliv.) Diels [Apiaceae, Angelicae sinensis radix]*,Paeonia veitchii* Lynch [Ranunculaceae, Paeoniae radix rubra],*Pheretima aspergillum (*E. Perrier) [Megascolecidae, Pheretima],*Ligusticum chuanxiong* Hort. [Apiaceae, Chuanxiong rhizoma],*Prunus persica* (L.) Batsch [Rosaceae, Persicae semen],*Carthamus tinctorius* L. [Asteraceae, Carthami flos]	Calycosin, Quercetin, Kaempferol, β-sitosterol and Stigmasterol	Protects retinal structures in RDD model	Not mentioned
22	Ma (2005)	Yishi-Tablet	*TuSiZi*, *ChuShiZi*, *ChongWeiZi*, *GouQiZi*, *QianRen*, *MuGua*, *HanShuiShi*, *ZiHeChe*, *SanQi*, *WuWeiZi*	*Cuscuta chinensis* Lam. [Convolvulaceae, Cuscutae semen]*,Broussonetia papyrifera* (L.) L'Hér. ex Vent. [Moraceae, Broussonetiae fructus],*Leonurus japonicus* Houtt. [Lamiaceae, Leonuri fructus],*Lycium chinense* Mill. [Solanaceae, Lycii fructus],*Plantago asiatica* L. [Plantaginaceae, Plantaginis semen],*Chaenomeles sinensis* (Thouin) Koehne [Rosaceae, Chaenomelis fructus],*Gypsum rubrum* [Mineral, Gypsum rubrum],*Homo sapiens* Linnaeus [Hominidae, Hominis placenta pulvis],*Panax notoginseng* (Burkill) F.H. Chen ex C.Y. Wu et K.M. Feng [Araliaceae, Notoginseng radix pulvis],*Schisandra chinensis* (Turcz.) Baill. [Schisandraceae, Schisandrae chinensis fructus]	Quercetin, β-sitosterol and Luteolin	Exhibits anti-apoptotic in RDD models	Affiliated Hospital of Chengdu University of Traditional Chinese Medicine
23	Ye et al. (2004)	Ming-Mu-Wu-Zi	*GouQiZi*, *TuSiZi*, *WuWeiZi*, *ChongWeiZi*, *ChuShiZi*	*Lycium chinense* Mill. [Solanaceae, Lycii fructus],*Cuscuta chinensis* Lam. [Convolvulaceae, Cuscutae semen],*Schisandra chinensis* (Turcz.) Baill. [Schisandraceae, Schisandrae chinensis fructus],*Leonurus japonicus* Houtt. [Lamiaceae, Leonuri fructus],*Broussonetia papyrifera* (L.) L'Hér. ex Vent. [Moraceae, Broussonetiae fructus]	Cycloartenol, Stigmasterol,β-Sitosterol,Sesamin, Isorhamnetin, Isofucosterol,Deoxyharringtonine, Oxyavicine	Exhibits anti-apoptotic in RDD model	Not mentioned
24	Yang (2003)	Yishi-Tablet	*TuSiZi*, C*huShiZi*, C*hongWeiZi*, G*ouQiZi*, *QianRen*, *MuGua*, *HanShuiShi*, *ZiHeChe*, *SanQi*, *WuWeiZi*	*Cuscuta chinensis Lam.* [Convolvulaceae, Cuscutae semen],*Broussonetia papyrifera* (L.) L'Hér. ex Vent. [Moraceae, Broussonetiae fructus],*Leonurus japonicus* Houtt. [Lamiaceae, Leonuri fructus],*Lycium chinense* Mill. [Solanaceae, Lycii fructus],*Plantago asiatica* L. [Plantaginaceae, Plantaginis semen],*Chaenomeles sinensis* (Thouin) Koehne [Rosaceae, Chaenomelis fructus],*Gypsum* rubrum [Mineral, Gypsum rubrum],*Homo sapiens* Linnaeus [Hominidae, Hominis placenta pulvis],*Panax notoginseng* (Burkill) F.H. Chen ex C.Y. Wu et K.M. Feng [Araliaceae, Notoginseng radix pulvis],*Schisandra chinensis* (Turcz.) Baill. [Schisandraceae, Schisandrae chinensis fructus]	Quercetin, β-sitosterol and Luteolin	Exhibits antioxidant stress effects, protects retinal functions in RDD model	Affiliated Hospital of Chengdu University of Traditional Chinese Medicine

As this study constitutes a systematic review of published literature, this study did not directly display the process of performing plant morphological authentication, DNA barcoding analysis, or voucher specimen deposition. The quality controls of the included TCM formulas followed the Pharmacopoeia of the People’s Republic of China (ChP) and Good Manufacturing Practice (GMP) regulations. As a systematic review and meta-analysis, this study does not involve the direct collection, processing, or trade of any plant material. The processing and preparation methods for the TCM formulas were documented in the original source studies.

### 3.3 Assessment of safety and adverse events

The assessment of safety and adverse events across the 24 included studies revealed that 10 studies mentioned the circumstances of animal exclusion due to adverse reactions. Six studies (Yao et al., 2012; Liu et al., 2024; Miao, 2023; Xu et al., 2016; Deng, 2010; Wang et al., 2011) reported animal mortality due to modeling-related complications or during the acclimatization period. One study (Fang, 2020) documented rat deaths caused by unexplained intestinal obstruction and progressive emaciation. Two studies (Cui, 2009; Yang, 2003) involved fatalities resulting from anesthetic overdose, while one study (Che, 2020) reported deaths associated with anterior segment inflammation post-modelling and abdominal distension following gavage administration. The included studies did not directly attribute animal mortality or specific adverse events to the administered TCM formulas. Most reported deaths appeared to be related to either the inherent severity of the disease model or procedural complications.

### 3.4 Meta-analysis results

This section of the meta-analysis evaluated the effects of TCM formulas in RDD models, with the aim of assessing the potential of TCM formulas to ameliorate neural factor expressions, apoptosis injuries, oxidative stress injuries, and retinal functional and structural indicators.

#### 3.4.1 Potential effects of TCM formulas on neural growth and glial activation indicators

**FIGURE 2:**
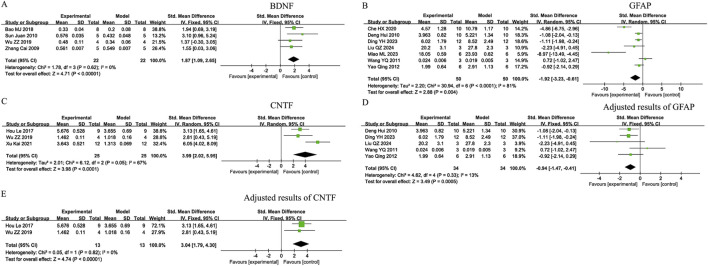
Forest plots of neural growth and glial activation indicators in Traditional Chinese Medicine(TCM) formulas. **(A)** Brain-derived neurotrophic factor (BDNF); **(B)** Glial fibrillary acidic protein (GFAP); **(C)** Ciliary Neurotrophic Factor (CNTF); **(D)** Adjusted results of GFAP; **(E)** Adjusted results of CNTF.

Owing to the low heterogeneity observed across the studies (I^2^ = 0% for BDNF), fixed-effects models were adopted for the meta-analyses. Zhen-Bao-Wan, Huo-Xue-Hua-Yu decoction, and Mi-Meng-Hua-Compound prescription led to significant increases in BDNF levels [SMD = 1.87; 95% CI: 1.09 to 2.65; Z = 4.71; P < 0.00001], as shown in Figure 2A.

Owing to the substantial heterogeneity observed in GFAP and CNTF (I^2^ = 81% and 67%, respectively), according to Figures 2B,C, sensitivity analyses were performed using STATASE 16 software to evaluate the robustness of each included trial for these outcomes. Sensitivity analyses revealed that heterogeneity in GFAP was primarily attributed to Che (2020) and Miao (2023), as shown in Figure 5A, while heterogeneity in CNTF was primarily attributed to Xu et al. (2021), as shown in Figure 5B. Detailed results of the sensitivity analyses are provided in Supplementary Data Sheet S2. Upon exclusion of these studies, the heterogeneity substantially decreased to 13% (GFAP) and 0% (CNTF), and the results revealed statistically significant reductions in GFAP expression (p < 0.05) after the administration of Qi-Shen-Yi-Qi pills, Qu-Yu-Tong-Luo prescription, Bu-Shen-Huo-Xue decoction, Zuo-Gui pill, and the Huo-Xue-Jie-Du recipe [SMD=−0.94; 95% CI: −1.47 to −0.41; Z = 3.49; *P* = 0.0005], as shown in Figure 2D. Statistical analysis revealed a significant increase in the CNTF levels following treatment with Bu-Shen-Yi-Jing-Fang and Mi-Meng-Hua -Compound prescriptions [SMD=3.04; 95% CI: 1.79 to 4.30; Z = 4.74; *P* < 0.00001], as shown in Figure 2E.

#### 3.4.2 Potential effects of TCM formulas on the regulation of oxidative stress and apoptotic indicators

**FIGURE 3:**
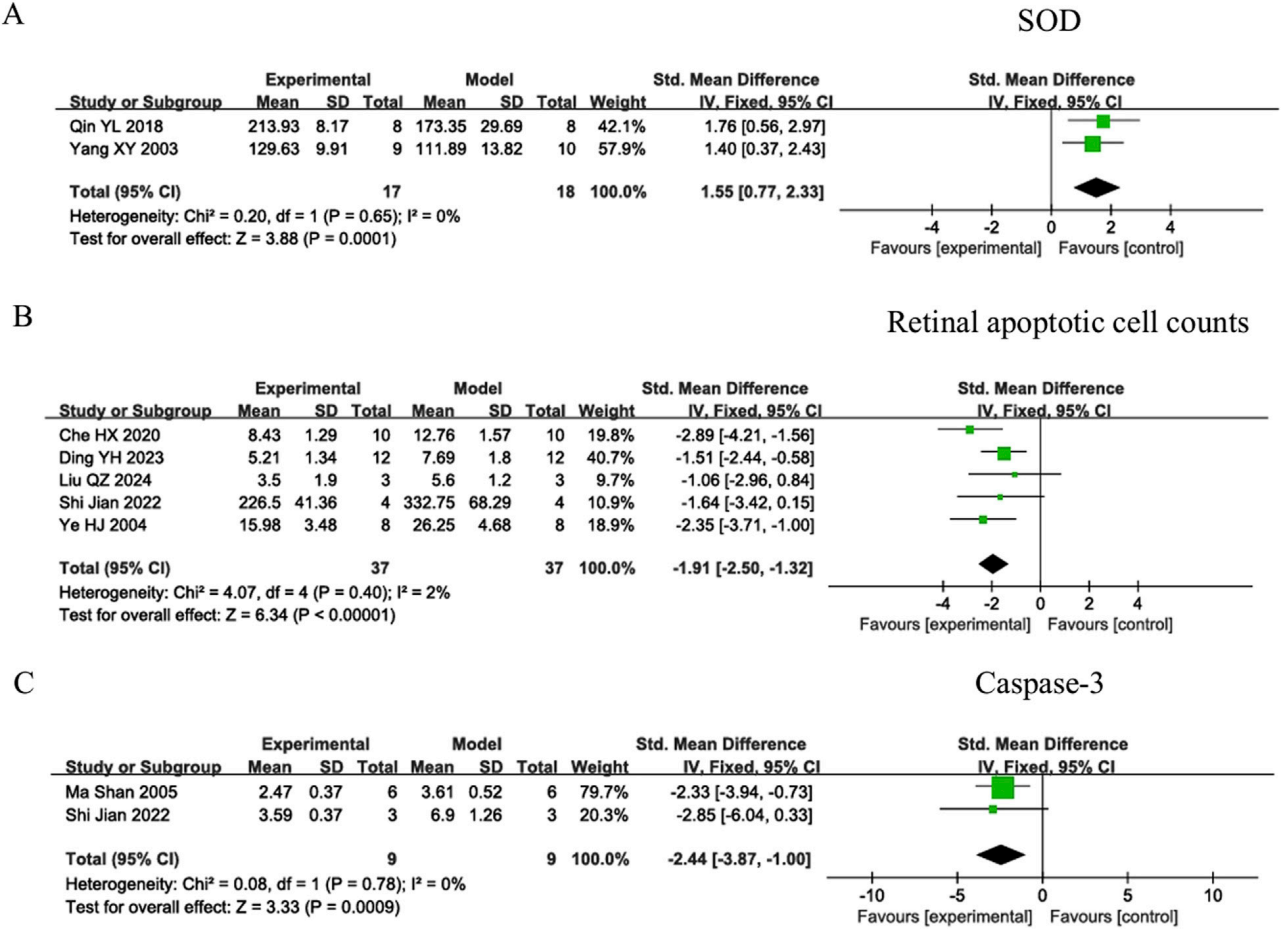
Forest plots of oxidative stress and apoptosis indicators in TCM formulas. **(A)** Superoxide dismutase (SOD); **(B)** Retinal apoptotic cell counts; **(C)** Cysteine-dependent aspartate-specific protease 3(Caspase-3).

Owing to the low heterogeneity observed across the studies (I^2^ = 0% for SOD, 2% for retinal apoptotic cell counts, and 0% for caspase-3), fixed-effects models were adopted for these meta-analyses. The Yi-Qi-Wen-Yang-Tong-Luo decoction and Yishi-Tablet significantly upregulated SOD expression [SMD = 1.55; 95% CI: 0.77 to 2.33; Z = 3.88; *P* = 0.0001], as shown in Figure 3A. Additionally, a marked decrease in retinal apoptotic cell counts was observed for the Zi-Yin-Ming-Mu decoction, Qu-Yu-Tong-Luo prescription, Bu-Shen-Huo-Xue decoction, Qing-Guang-An granule, and Ming-Mu-Wu-Zi prescription [SMD = −1.91; 95% CI: −2.50 to −1.32; Z = 6.34; *P* < 0.00001], as shown in Figure 3B. Furthermore, administration of Yishi-Tablet and Qing-Guang-An granule was associated with pronounced decreases in caspase-3 activity [SMD = −2.44; 95% CI: −3.87 to −1.00; Z = 3.33; *P* = 0.0009], as shown in Figure 3C.

#### 3.4.3 Potential effects of TCM formulas on the regulation of ophthalmological indicators

**FIGURE 4:**
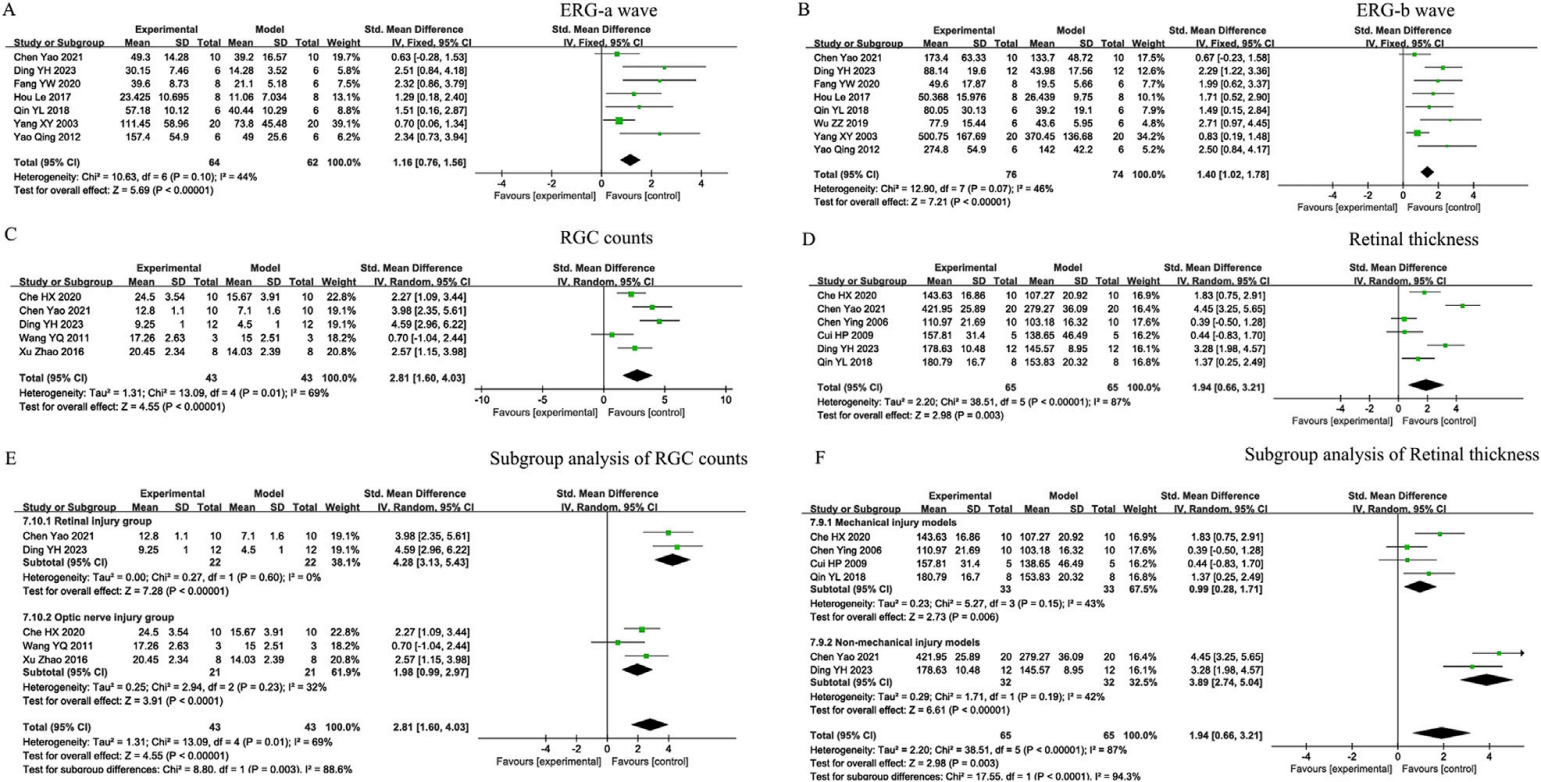
Forest plots of ophthalmological indicators in TCM formulas. **(A)** ERG-a wave amplitude; **(B)** ERG-b wave amplitude; **(C)** Retinal ganglion cell (RGC) counts; **(D)** Retinal thickness; **(E)** Subgroup analysis of RGC counts; **(F)** Subgroup analysis of Retinal thickness.

Owing to the low heterogeneity observed in the ERG-a wave (44%) and ERG-b wave (46%) analyses, fixed-model effects were performed to analyse these outcomes. The Bu-Yang-Huan-Wu decoction, Qu-Yu-Tong-Luo prescription, Ming-Mu-Xiao-Meng tablet, Bu-Shen-Yi-Jing-Fang, Yi-Qi-Wen-Yang-Tong-Luo decoction, and Yishi-Tablet treatments resulted in significantly higher levels of the ERG-a wave (SMD = 1.16; 95% CI: 0.76 to 1.56; Z = 5.69; *P* < 0.00001), as shown in Figure 4A. Similarly, the Bu-Yang-Huan-Wu decoction, Qu-Yu-Tong-Luo prescription, Ming-Mu-Xiao-Meng tablet, Bu-Shen-Yi-Jing-Fang, Yi-Qi-Wen-Yang-Tong-Luo decoction, Yishi-Tablet, and Mi-Meng-Hua-Compound prescription treatments resulted in significantly higher levels of ERG-b wave [SMD=1.40, 95% CI: 1.02 to 1.78; Z = 7.21; *P* < 0.00001], as shown in Figure 4B.

**FIGURE 5:**
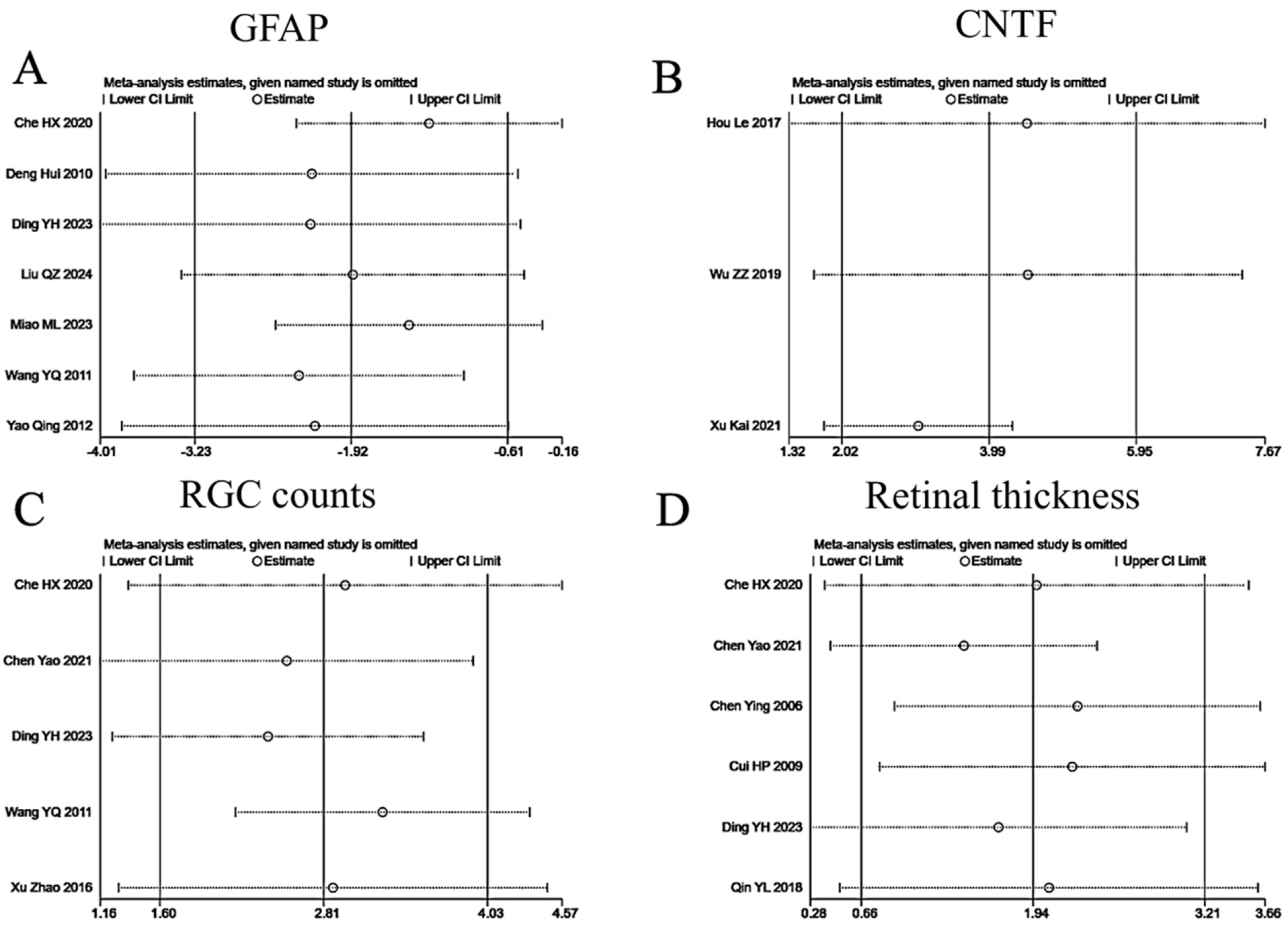
Robustness assessment through sensitivity analysis. **(A)** GFAP; **(B)** CNTF; **(C)** RGC counts; **(D)** Retinal thickness.

Owing to the substantial heterogeneity observed in the RGC counts (I^2^ = 69%) and retinal thickness (I^2^ = 87%), according to Figures 4C,D, sensitivity analyses were performed using STATASE 16 software to evaluate the robustness of each included trial for these outcomes. Sensitivity analyses revealed robust estimates of RGC counts (Figure 5C, Supplementary Data Sheet S2) and retinal thickness (Figure 5D, Supplementary Data Sheet S2) in the sequential exclusion analysis, indicating that the heterogeneity was not driven by any individual study. Subgroups were further analysed to determine the sources of heterogeneity.

#### 3.4.4 Subgroup analyses for high heterogeneity outcomes (RGC counts and retinal thickness)

To investigate whether TCM formulas have specific effects on the RGC counts and retinal thickness through the use of different methods or distinct primary sites of pathological damage, we conducted subgroup analyses on the basis of the primary site of pathological damage or the methods used to construct disease models.

First, two studies in which retinal injuries were established demonstrated that the RGC counts were significantly greater after the administration of the Bu-Yang-Huan-Wu decoction and Qu-Yu-Tong-Luo prescription [SMD = 4.28; 95% CI: 3.13 to 5.43; Z = 7.28; *P* < 0.00001], as shown in Figure 4E. Three studies that established optic nerve injuries demonstrated that the RGC counts were significantly greater after the administration of the Zi-Yin-Ming-Mu decoction, Zuo-Gui pill, and Xue-Fu-Zhu-Yu decoction (SMD = 1.98; 95% CI: 0.99 to 2.97; Z = 3.91; *P* < 0.0001), revealing that TCM formulas exhibited superior efficacy in retinal injuries models, as shown in Figure 4E.

Second, three studies in which mechanical injury models were established indicated that retinal thickness significantly increased after the administration of the Zi-Yin-Ming-Mu decoction, Bu-Yang-Huan-Wu decoction, Xue-Fu-Zhu-Yu decoction, and Yi-Qi-Wen-Yang-Tong-Luo decoction (SMD = 0.99; 95% CI: 0.28 to 1.71; Z = 2.73; *P* = 0.006), as shown in Figure 4F. Three studies in which nonmechanical injury models were established indicated that retinal thickness significantly increased after the administration of the Bu-Yang-Huan-Wu decoction and Qu-Yu-Tong-Luo prescription [SMD = 3.89; 95% CI: 2.74 to 5.04; Z = 6.61; *P* < 0.00001], revealing that TCM formulas exhibited superior efficacy in nonmechanical injury, as shown in Figure 4F.

### 3.5 Bayesian network meta-analysis

#### 3.5.1 The results of the transitivity assumption and consistency assumption

The transitivity assumption (Hou, 2017; Xu et al., 2021; Chen et al., 2006; Chen, 2021; Sun, 2010; Zhang, 2009; Yao et al., 2012; Liu et al., 2024; Ye et al., 2004; Fang, 2020; Shi et al., 2022a; Shi et al., 2022b; Miao, 2023; Wu et al., 2019; Cui, 2009; Xu et al., 2016; Deng, 2010; Qin, 2018; Ma, 2005; Yang, 2003; Bao et al., 2018; Che, 2020; Ding, 2022; Wang et al., 2011) was assessed through examination of the distribution of potential effect modifiers across treatment comparisons within the network. Multiple RDD models and varied modelling approaches were employed across included studies, which may introduce heterogeneity. However, retinal injury and functional loss were consistently demonstrated in all studies, with neuronal apoptosis leading to degenerative retinopathy being established as the definitive endpoint. Furthermore, all outcomes were assessed using standardized and widely accepted techniques without severe systematic bias being identified. Although methodological heterogeneity was observed, violation of the transitivity assumption was not supported. Detailed characteristics of all included studies are presented in Table 1.

The global consistency assumptions (Hou, 2017; Xu et al., 2021; Chen et al., 2006; Chen, 2021; Sun, 2010; Zhang, 2009; Yao et al., 2012; Liu et al., 2024; Ye et al., 2004; Fang, 2020; Shi et al., 2022a; Shi et al., 2022b; Miao, 2023; Wu et al., 2019; Cui, 2009; Xu et al., 2016; Deng, 2010; Qin, 2018; Ma, 2005; Yang, 2003; Bao et al., 2018; Che, 2020; Ding, 2022; Wang et al., 2011) in comparison between consistency and inconsistency models exhibited ΔDIC<5 in all included outcomes, suggesting reasonable agreement between direct and indirect evidence. An assessment of local inconsistency via node-splitting was not applicable due to the network topology, which did not contain the necessary independent direct and indirect evidence loops for any specific comparison. Detailed results are shown in Supplementary Table S3, and the raw analysis data from R is documented in Supplementary Data Sheet S7. Supplementary Data Sheet S5 contains forest plots illustrating treatment effects compared to the model group, which are consistent with the results from pairwise meta-analyses.

#### 3.5.2 Results of the NMA network

**FIGURE 6:**
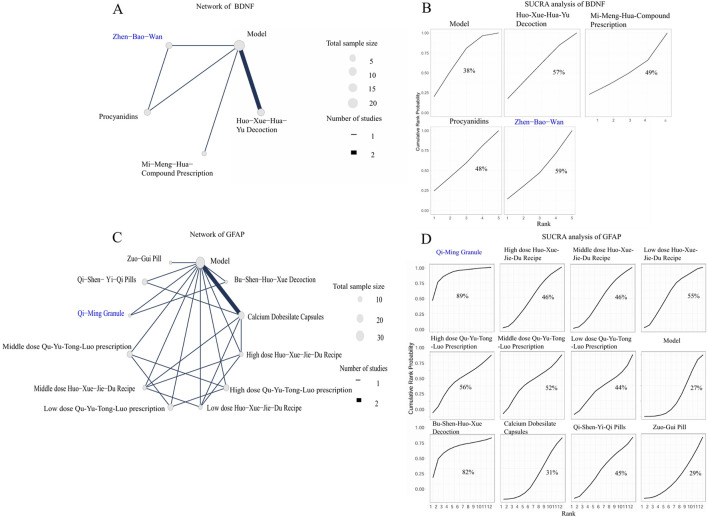
The network meta-analysis (NMA) analyses of BDNF and GFAP. Nodes represent treatments, and the connecting lines indicate available direct comparisons. The size of the nodes is proportional to the total sample size for each intervention. The percentage values represent the surface under the cumulative ranking curve. A higher surface under the cumulative ranking curve (SUCRA) value indicates a trend of being greater probability of that intervention being among the best options. The intervention with the highest SUCRA value is highlighted in blue in both the network and SUCRA plots. **(A)** Network of BDNF; **(B)** SUCRA analysis of BDNF; **(C)** Network of GFAP; **(D)** SUCRA analysis of GFAP.

**FIGURE 7:**
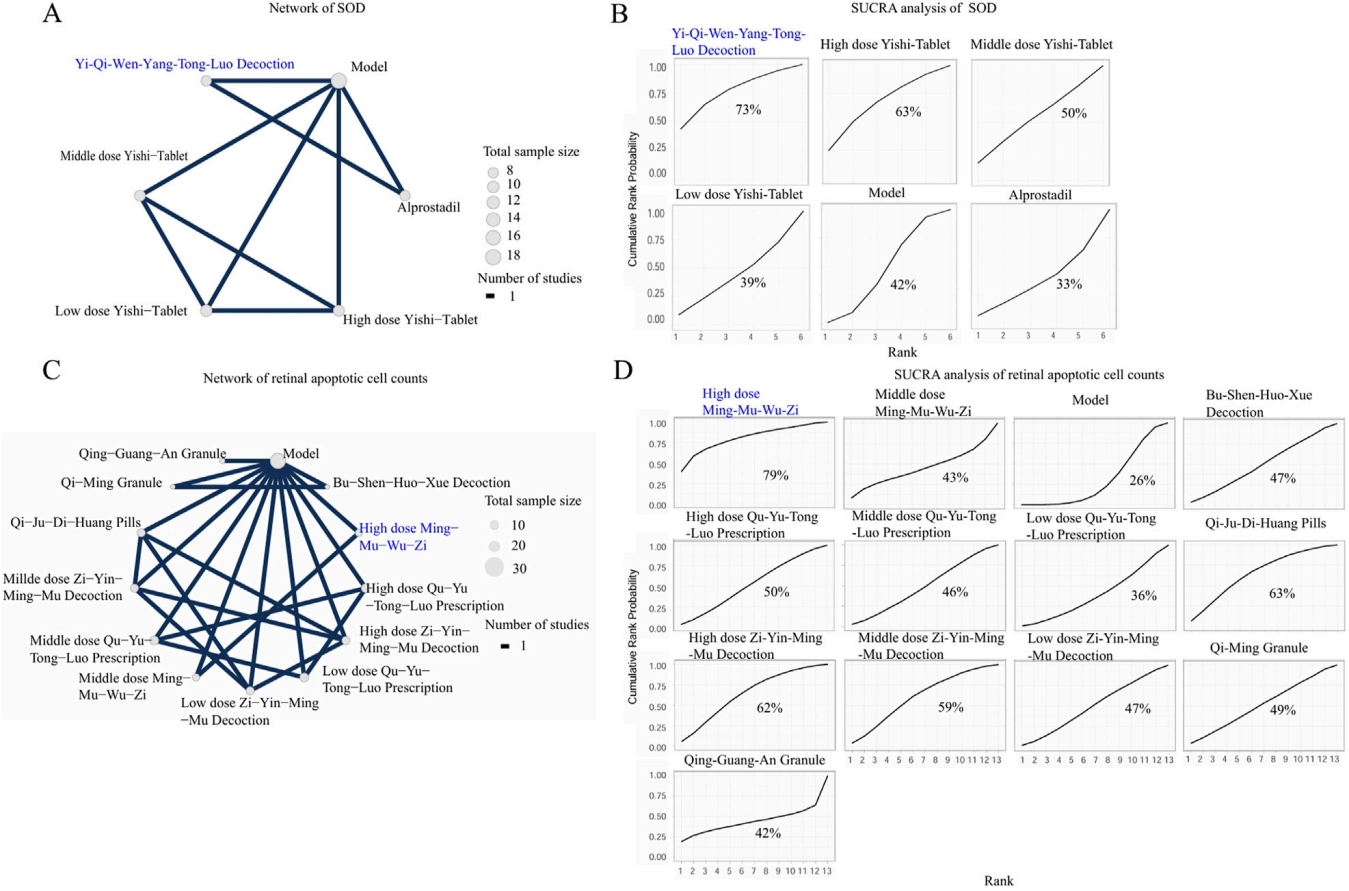
The NMA analyses of SOD and retinal apoptotic cell counts. Nodes represent treatments, and the connecting lines indicate available direct comparisons. The size of the nodes is proportional to the total sample size for each intervention. The percentage values represent the surface under the cumulative ranking curve. A higher SUCRA value indicates a trend of being greater probability of that intervention being among the best options. The intervention with the highest SUCRA value is highlighted in blue in both the network and SUCRA plots. **(A)** Network of SOD; **(B)** SUCRA analysis of SOD; **(C)** Network of retinal apoptotic cell counts; **(D)** SUCRA analysis of retinal apoptotic cell counts.

**FIGURE 8:**
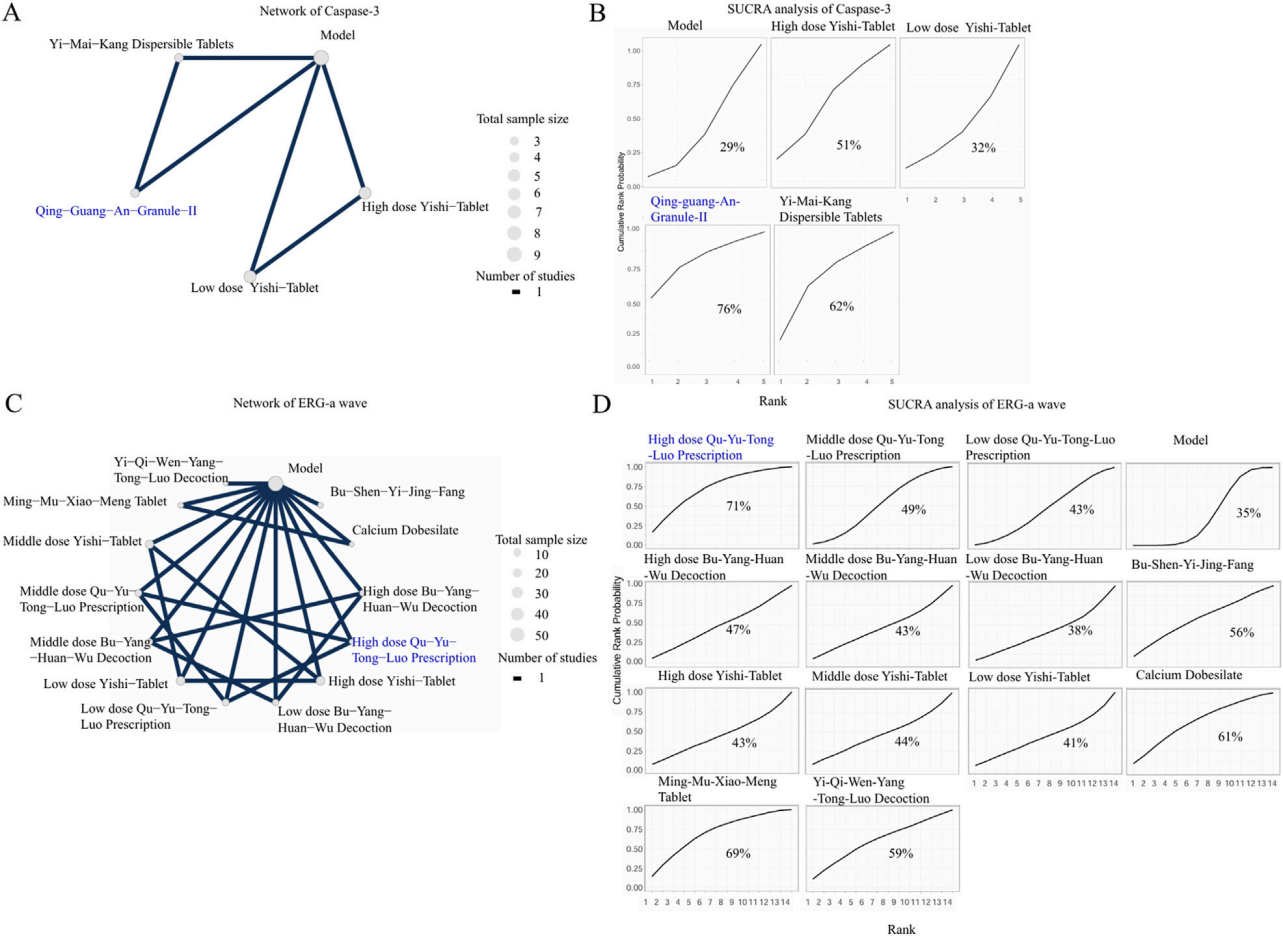
The NMA analyses of Caspase-3 and ERG-a wave. Nodes represent treatments, and the connecting lines indicate available direct comparisons. The size of the nodes is proportional to the total sample size for each intervention. The percentage values represent the surface under the cumulative ranking curve. A higher SUCRA value indicates a trend of being greater probability of that intervention being among the best options. The intervention with the highest SUCRA value is highlighted in blue in both the network and SUCRA plots. **(A)** Network of Caspase-3; **(B)** SUCRA analysis of Caspase-3; **(C)** Network of ERG-a wave; **(D)** SUCRA analysis of ERG-a wave.

**FIGURE 9:**
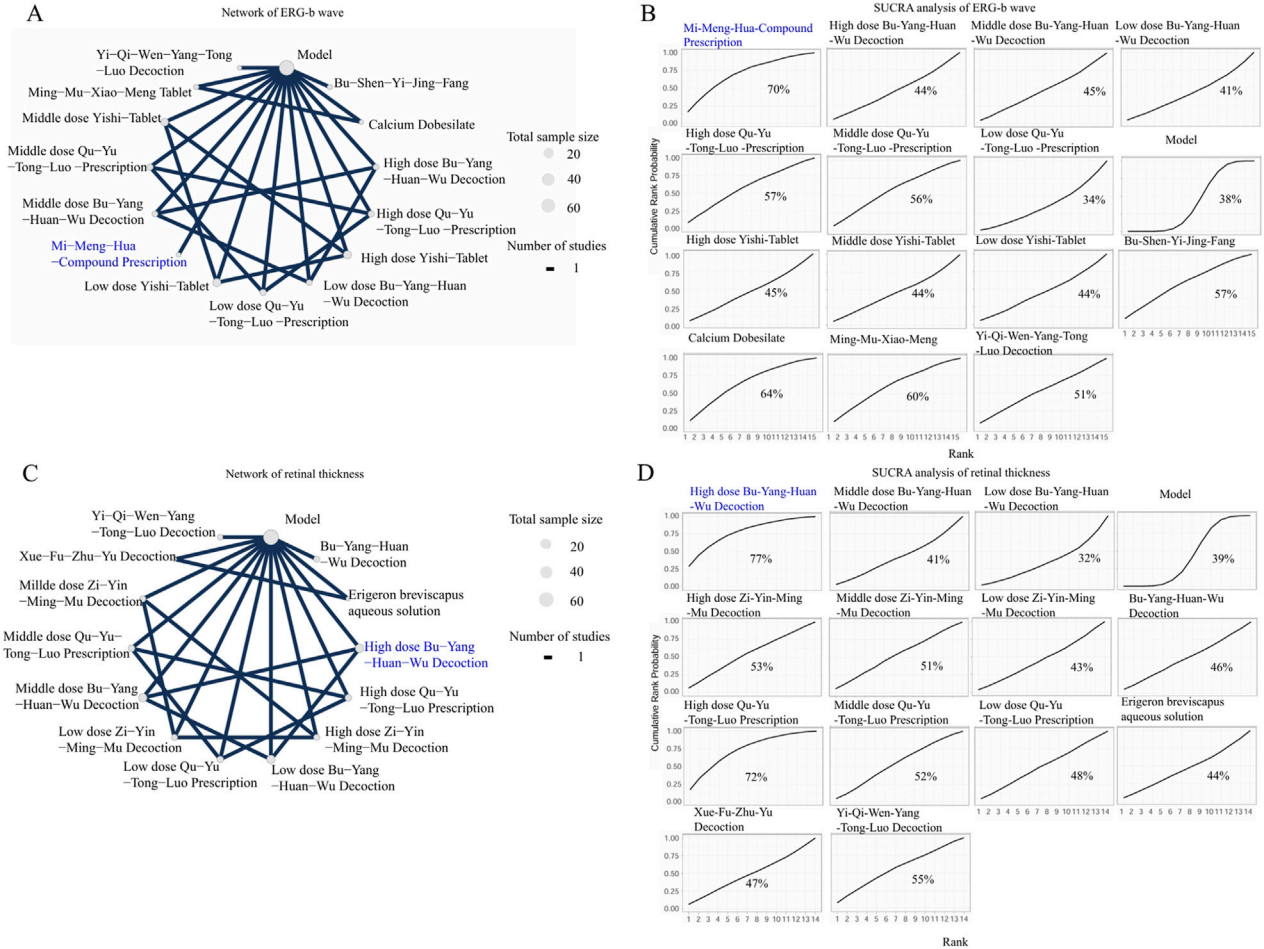
The NMA analyses of ERG-b wave and retinal thickness. Nodes represent treatments, and the connecting lines indicate available direct comparisons. The size of the nodes is proportional to the total sample size for each intervention. The percentage values represent the surface under the cumulative ranking curve. A higher SUCRA value indicates a trend of being greater probability of that intervention being among the best options. The intervention with the highest SUCRA value is highlighted in blue in both the network and SUCRA plots. **(A)** Network of ERG-b wave; **(B)** SUCRA analysis of ERG-b wave; **(C)** Network of retinal thickness; **(D)** SUCRA analysis of retinal thickness.

**FIGURE 10:**
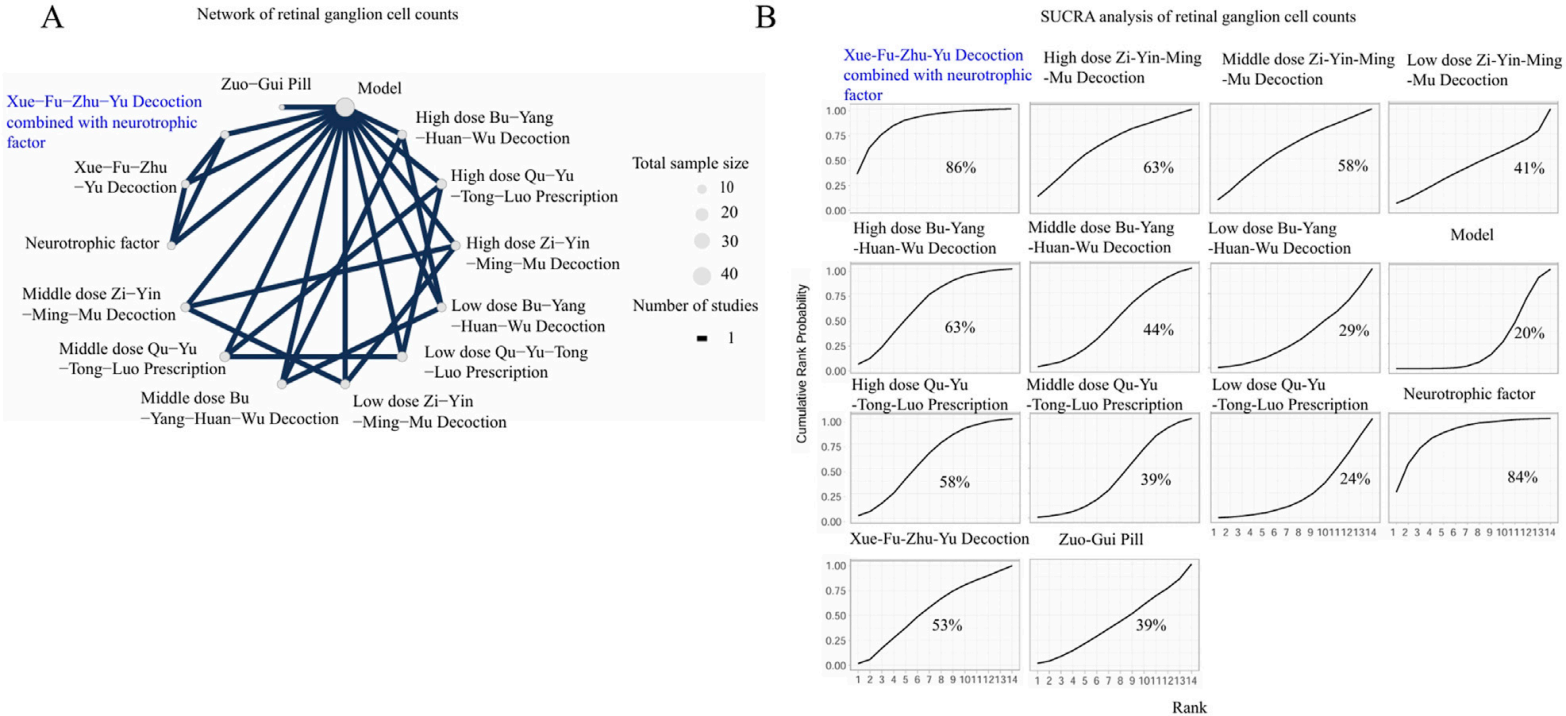
The NMA analyses of RGC counts. Nodes represent treatments, and the connecting lines indicate available direct comparisons. The size of the nodes is proportional to the total sample size for each intervention. The percentage values represent the surface under the cumulative ranking curve. A higher SUCRA value indicates a trend of being greater probability of that intervention being among the best options. The intervention with the highest SUCRA value is highlighted in blue in both the network and SUCRA plots. **(A)** Network of RGC counts. **(B)** SUCRA analysis of number of RGC counts.

- The network of BDNF incorporated four interventions: Zhen-Bao-Wan, Procyanidins, the Huo-Xue-Hua-Yu decoction, and Mi-Meng-Hua compound prescription, with the model group serving as the common comparator. As depicted in Figure 6A, the network was small and sparse, consisting of only four interventions with no closed loops of direct comparisons, increasing the uncertainty in estimating their relative rankings. As depicted in Figure 6B and Supplementary Data Sheet S6, the SUCRA analysis indicated a trend that Zhen-Bao-Wan was one of the higher-ranking interventions for elevating BDNF, with an estimated ranking probability of around 59%. However, the evidence is not conclusive, indicating that the ranking results for BDNF should be viewed as preliminary and hypothesis-generating. Future studies targeting TCM formulas for BDNF are needed to confirm these findings.
- The network of GFAP, consisting of 11 treatments from 5 studies, incorporated a more complex and well-connected network, as illustrated in Figure 6C. This network included different doses of TCM formulas, biomedicine controls, and a sham-injury control group, indicating the presence of both direct and indirect evidence for several comparisons. The size of the nodes and the thickness of the connecting lines revealed variations in total sample size and the number of studies that were available for specific comparisons. As depicted in Figure 6D and Supplementary Data Sheet S6, the SUCRA analysis showed a trend that Qi-Ming granules could potentially be one of the more effective options for GFAP modulation, with an estimated ranking probability of around 89%.
- The network of SOD incorporated data from 2 studies involving 5 treatments and revealed a partially connected topology, as depicted in Figure 7A. These included three dosage levels (low, middle, and high) of Yishi-Tablet, Yi-Qi-Wen-Yang-Tong-Luo decoction, and the positive control biomedicines. A direct comparison loop existed among the different doses of Yishi-Tablet, providing relative efficacy estimates within this specific formulation. As depicted in Figure 7B and Supplementary Data Sheet S6, the SUCRA analysis showed a trend that the Yi-Qi-Wen-Yang-Tong-Luo decoction could exhibit the greatest likelihood of modulating SOD activity, with an estimated ranking probability of around 73%.
- The network of retinal apoptotic cell counts integrated data from 5 studies involving 12 treatments and incorporated a densely connected evidence network, as presented in Figure 7C. The included treatments included multiple TCM formulas across various dosage regimens (e.g., high-dose Zi-Yin-Ming-Mu decoction, middle-dose Zi-Yin-Ming-Mu decoction, and low-dose Zi-Yin-Ming-Mu decoction), in addition to standalone formulas such as the Bu-Shen-Huo-Xue decoction. As depicted in Figure 7D and Supplementary Data Sheet S6, SUCRA analysis showed a trend that high-dose Ming-Mu-Wu-Zi was one of the most favourable treatments for reducing the retinal apoptotic cell counts, with an estimated ranking probability of around 79%.
- The network of caspase-3 incorporated data from 2 studies, encompassing 4 active interventions—high-dose Yishi-Tablet, low-dose Yishi-Tablet, Qing-Guang-An-Granules-II, and Yi-Mai-Kang dispersible tablets—exhibiting a limited and sparsely connected evidence structure, as shown in Figure 8A. The network topology exhibited a star-shaped feature, introducing uncertainty regarding the comparative efficacies and efficiencies of the interventions. The results of the SUCRA analysis (76%) suggested a trend that Qing-Guang-An-Granule-II was one of the highest-ranking interventions for reducing Caspase-3 expression, shown in Figure 8B and Supplementary Data Sheet S6.
- The network of ERG-a waves incorporated data from 6 studies in 13 treatments and revealed a well-developed and connected network, as illustrated in Figure 8C. The interventions included multiple dosages of TCM formulas, positive control biomedicine. The network topology was characterized by its complexity, featuring multiple closed loops and thick connecting lines. This finding indicates a robust body of evidence with numerous direct comparisons among the interventions, which strengthens the reliability of the relative effect estimates. The results of SUCRA analysis (71%) showed a trend that high-dose Qu-Yu-Tong-Luo prescription indicated a potential advantage for ERG-a modulation, shown in Figure 8D and Supplementary Data Sheet S6.
- The network of ERG-b wave incorporated 7 studies in 14 treatments and revealed a large and intricately connected evidence network, as depicted in Figure 9A. This network included multiple dosages of TCM formulas (e.g., high-dose Qu-Yu-Tong-Luo prescription, middle-dose Qu-Yu-Tong-Luo prescription), and positive control biomedicines. The network topology demonstrated high complexity, with numerous thick connecting lines and closed loops, indicating a substantial body of direct evidence comparisons. This dense connectivity greatly enhances the reliability and precision of the relative efficacy estimates. The results of SUCRA analysis (70%) showed a trend that the Mi-Meng-Hua-Compound prescription indicated a potential advantage for ERG-b wave modulation, shown in Figure 9B and Supplementary Data Sheet S6.
- The network of the retinal thickness incorporated data from 6 studies involving 13 treatments, as shown in Figure 9C. The network included various TCM formulas, such as the high-dose Bu-Yang-Huan-Wu decoction, high-dose Qu-Yu-Tong-Luo prescription, positive control biomedicines. This network featured several direct comparison loops, particularly between different doses of the same formula, exhibiting a moderate level of connectivity. The results SUCRA analysis (77%) showed a trend that the high-dose Bu-Yang-Huan-Wu decoction was one of the most effective treatments for increasing retinal thickness, shown in Figure 9D and Supplementary Data Sheet S6.
- The network of the RGC counts network incorporated well-connected data from 5 studies in 13 treatments, demonstrating multiple direct comparison loops and connections with varying thicknesses (Figure 10A). These strategies included a range of TCM formulas (e.g., high-dose Zi-Yin-Ming-Mu decoction and middle-dose Zi-Yin-Ming-Mu decoction), positive control biomedicines. The results of the SUCRA analysis (86%) showed a trend that the Xue-Fu-Zhu-Yu decoction combined with neurotrophic factor was among the most effective treatments for restoring the RGC counts, shown in Figure 10B and Supplementary Data Sheet S6.

### 3.6 Publication bias analysis

For the majority of outcomes, both the Begg’s and Egger’s tests indicated no statistically significant evidence of publication bias (*P* ≥ 0.05), including the RGC counts, GFAP, caspase-3, BDNF, CNTF, retinal apoptotic cell counts, retinal thickness, but potential publications bias were detected for the ERG-a wave and ERG-b wave, where Begg’s or/and Egger’s tests yielded the p values<0.05. The detailed results of the Begg’s test Egger’s tests are comprehensively summarized in Supplementary Table S1 and Supplementary Data Sheet S3. Further assessment of potential publication bias for the ERG-a wave and ERG-b wave outcomes were conducted using the trim and fill method. The trim and fill analysis analyses suggest that the addition of three studies for ERG-a wave and four studies for ERG-b wave would be theoretically required to achieve the funnel plot symmetry. The imputation of these studies did not substantially alter the direction or statistical significance of the pooled effect estimate, indicating the robustness of our primary finding. The detailed process of trim and fill analysis are provided in the Supplementary Data Sheet S4.

### 3.7 SYRCLE assessment results

The methodological quality of the 24 included animal studies, assessed via SYRCLE’s risk of bias criteria (Hooijmans et al., 2014), revealed pervasive limitations in terms of randomization, blinding, and outcome assessment protocols. These limitations necessitate a cautious interpretation of the meta-analysis results. Efforts to improve the adherence to standardized reporting guidelines are urgently needed to enhance the reliability of future preclinical studies. The results of the SYRCLE assessment are comprehensively summarized in Supplementary Table S2.

## 4 Discussion

By synthesizing evidence from 24 preclinical studies, this study demonstrated that specific TCM formulas showed signals of neuroprotective potential in animal models of RDDs. By synthesizing existing studies and critically appraising their quality, this study serves as exploratory research reflecting the current state of evidence in this field, which demonstrates promising biological signals but requires methodological improvements in future investigations.

First, this study revealed that Zhen-Bao-Wan, Huo-Xue-Hua-Yu decoction, and Mi-Meng-Hua-Compound prescription significantly increased BDNF while Bu-Shen-Yi-Jing-Fang and Mi-Meng-Hua-Compound prescriptions enhanced CNTF. Among the analysed TCM formulas, β-sitosterol and stigmasterol were identified as the shared core bioactive compounds associated with BDNF enhancement, while luteolin was the common compound associated with CNTF improvement. β-sitosterol has been reported to modulate BDNF expression and neurotrophic signalling pathways through enhanced neuronal survival, synaptic plasticity, and HPA axis normalization, contributing to neuroprotection and stress resilience (Sen et al., 2025; Xu and Yu, 2025). Stigmasterol has been proved to effectively alleviate high glucose-induced cell damage and DR by suppressing ROS, apoptosis, and VAMP7-mediated autophagy (Wang et al., 2025). Luteolin has been shown to demonstrate neuroprotective effects in autoimmune encephalomyelitis by upregulating CNTF expression (El-Deeb et al., 2019). The increased levels of BDNF and CNTF suggest that bioactive compounds such as β-sitosterol, stigmasterol and Luteolin could promote neuronal survival and synaptic plasticity, potentially via the BDNF/CNTF signalling pathway. While elevated BDNF and CNTF levels correlate with these TCM formulas and their key bioactive compounds, their causal roles require further mechanistic validation. Future work should investigate the temporal dynamics of BDNF/CNTF coactivation to optimize targeted therapeutic strategies.

Second, this study demonstrated that Qi-Shen-Yi-Qi pills, Qu-Yu-Tong-Luo prescription, Bu-Shen-Huo-Xue decoction, Zuo-Gui pill, and Huo-Xue-Jie-Du recipe could inhibit glial activation by reducing GFAP expression following retinal stress and injury, which creates a more favourable microenvironment for neuronal recovery. Among these analysed TCM formulas, quercetin, cryptotanshinone, kaempferol, sitosterol, stigmasterol, ginsenosides were identified as the shared bioactive compounds correlated with GFAP modulation. As one of the flavonoids, quercetin has been reported to exert neuroprotective effects by promoting beneficial astrocyte activation and suppressing detrimental JAK2/STAT3 pathways (Wang Y. et al., 2018), while also inhibiting LED-induced GFAP upregulation in the retina, highlighting its dual role in modulating astrocyte responses (Sahin et al., 2023). Cryptotanshinone has been identified as an active component in Bu-Shen-Huo-Xue Prescriptions that negatively correlates with VEGF and PKC-β expression in Müller cells, suggesting its potential role in modulating GFAP-associated glial activation in DR (Yu et al., 2023). β-sitosterol-β-D-glucoside has been reported to induce GFAP+/C3+ neurotoxic A1 reactive astrocyte activation alongside chronic neuroinflammation and dopaminergic neurodegeneration (Luna-Herrera et al., 2020). Ginsenosides, particularly ginsenoside Ro, Rb1, Rg1, Rg2, and F1, have been reported to exhibit neuroprotective effects by reducing GFAP-positive astrocyte activation and modulating the MAPK and TLR4/NF-κB pathways (Li T. et al., 2025; Liu X. et al., 2025; Hou et al., 2022). Although these formulas and their bioactive compounds exhibit GFAP-reducing effects, their direct influence on GFAP expression remains to be experimentally validate. A deeper exploration of GFAP-regulated pathways is needed to understand their therapeutic potential for RDDs.

Furthermore, this study demonstrated that Yi-Qi-Wen-Yang-Tong-Luo decoction and Yishi-Tablet mitigate oxidative damage by modulating the activities of antioxidant enzymes such as SOD, thereby enhancing the endogenous antioxidant capacity. Among these analysed TCM formulas, sitosterol was identified as the shared bioactive compounds correlated with SOD modulation. β-Sitosterol, as a core active component of various formulations, has been reported to enhance SOD activity in diabetic kidney disease rats and restore SOD expression in liver injury models, demonstrating its critical antioxidant role through modulation of oxidative stress pathways (Devaraj et al., 2020; Han et al., 2025). β-Sitosterol was identified as one of the active compounds in Ginkgo biloba extract that may protect RGCs in open-angle glaucoma by modulating p53/Bcl-2/Bax/Caspase pathways to reduce oxidative stress-induced apoptosis (Yu et al., 2022). In addition, recent studies indicated that Lycii Fructus and Chrysanthemum Flos attenuate retinal oxidative injury and suppress apoptotic processes in Müller cells via dual modulation of the Nrf2/HO-1 pathway, which serves as a master regulator of cellular antioxidant defence (Cao et al., 2025). Li X. et al. (2025b) revealed that the Zi-yin-Ming-Mu decoction could protect against AMD through cholesterol level control, oxidative stress and inflammation mitigation, as well as through the regulation of the gut microbiota via its polyphenolic components. However, the precise causal relationship between these shared compounds and the formulas’ overall therapeutic effects requires further elucidation. Future studies should investigate the pharmacological contributions of these core compounds and delineate their potential synergistic mechanisms within the holistic context of TCM formulas.

Moreover, this study indicated that Zi-Yin-Ming-Mu decoction, Qu-Yu-Tong-Luo prescription, Bu-Shen-Huo-Xue decoction, Qing-Guang-An granule, and Ming-Mu-Wu-Zi prescription contributing to reducing retinal apoptotic cell counts. Besides, Yishi-Tablet and Qing-Guang-An granule were also found to downregulate the expression of the key apoptosis protein caspase-3. Among the analysed TCM formulas, quercetin, kaempferol, formononetin, β-sitosterol, stigmasterol, baicalein, cycloartenol, isorhamnetin, sesamin were identified as the shared core bioactive compounds associated with reducing retinal apoptotic cell counts, while quercetin luteolin and β-sitosterol were the common compound correlated with alleviating caspase-3. Quercetin has been proved to protect photoreceptors from retinal induced apoptosis by suppressing ROS generation and inhibiting ER stress, highlighting its therapeutic potential for RDDs such as dry AMD and Stargardt disease type 1 (Yang et al., 2024). Kaempferol has been found to exert anti-apoptotic effects in DR by modulating critical targets such as CASP3 and BCL2, thereby protecting retinal cells and contributing to the therapeutic efficacy of DR progression (Yang et al., 2025). Formononetin has been reported to prevent retinal apoptosis via PI3K/AKT/mTOR activation and oxidative stress/NF-κB inhibition, offering therapeutic potential for diabetic retinopathy (Li et al., 2023). Evidence indicates that β-Sitosterol and stigmasterol could protect RGCs from apoptosis in open-angle glaucoma by modulating p53 signalling, reducing Bax/Bcl-2 ratio, and inhibiting caspase-3/9 activation, demonstrating therapeutic potential in retinal degeneration (Wang et al., 2025; Yu et al., 2022; Yang et al., 2025). Evidence indicated that baicalein exerted neuroprotective effects against glaucoma by reducing intraocular pressure, suppressing RGC apoptosis, and modulating inflammatory cytokines, suggesting a therapeutic mechanism involving integrated regulation of apoptosis and inflammation (Yang et al., 2021). Previous studies has demonstrated that isorhamnetin protected retinal pigment epithelium cells from apoptosis by suppressing ROS production and caspase-3 activation, suggesting its therapeutic potential for AMD prevention (Wang J. et al., 2018). Sesamin has been reported to attenuate DR progression by reducing hyperglycaemia, suppressing microglial activation, and downregulating pro-inflammatory mediators, thereby protecting against retinal apoptosis in STZ-induced diabetic mice (Ahmad et al., 2016). These findings collectively underscore the importance of apoptotic pathways in retinal injury, highlighting potential therapeutic targets for neuroprotective interventions in RDDs. However, the current evidence predominantly relies on preclinical models and retrospective analyses, leaving the clinical efficacy and optimal dosing regimens of these compounds and formulas insufficiently validated in human populations. Future research should prioritize well-designed clinical trials to translate these mechanistic findings into tangible therapies, and further explore the synergistic interactions among the multi-component systems of TCM formulas.

Notably, the subgroup analyses and sensitivity analyses conducted in this study explained some of the sources of heterogeneity, particularly regarding the differential efficacy of TCM formulas on the basis of the primary site of pathology and injury mechanism. Our subgroup analyses revealed that Bu-Yang-Huan-Wu decoction and Qu-Yu-Tong-Luo prescription demonstrated favourable efficacy in restoring ganglion cell counts in retinal injury models and likewise improved retinal thickness in nonmechanical injury models, suggesting that Bu-Yang-Huan-Wu decoction and Qu-Yu-Tong-Luo prescription might exert their therapeutic effects mainly through preserving vascular integrity, protecting photoreceptors, attenuating inflammation, and alleviating oxidative stress. However, the exploration of the sources of high heterogeneity, such as retinal thickness (I^2^ = 87%), could be deepened. First, differences in disease models are key contributors. The different RDD models encompass diverse aetiologies, such as retinal vein occlusion, retinal light damage, and DR, each of which involve distinct pathophysiological processes that affect retinal structure differently. Second, disparities in measurement techniques contribute to the variability. The specific retinal layers measured and potential tissue processing artefacts might influence the absolute values and variability of the results. More importantly, variations in TCM formula composition, dosage, preparation methods, and administration routes are important for accounting for the sources of heterogeneity. This lack of standardization is inherent to much of traditional medicine research and reflects the personalized, syndrome-differentiated nature of TCM practice. Although the specific impact of these variations could not be quantified directly in our analysis, they introduce a potential source of variability that may have influenced the pooled effect estimates. Despite this, consistent neuroprotective effects emerged in this study, reinforcing the biological plausibility of the overall findings. Therefore, our results should be interpreted as evidence supporting the overall therapeutic potential of TCM formula-based strategies rather than as a definitive indication of any specific, unalterable formula. In future work, bridging the gap between traditional practice and modern translational science is essential. Future efforts should focus on establishing consensus guidelines for the quality control and standardization of key TCM formula parameters, such as the use of chemically characterized extracts, the definition of dose-equivalence metrics, and the adoption of rigorous reporting standards. These steps are critical for minimizing heterogeneity, enhancing the reproducibility of preclinical research, and paves the way for the development of standardized, evidence-based herbal products suitable for clinical evaluation.

Additionally, our NMA exhibited target-specific and dose‒response trends, with different formulas showing preferential efficacy for distinct biomarkers. For instance, Qi-Ming granules (SUCRA = 89%) were one of the most effective choices for suppressing GFAP, whereas Qing-Guang-An-Granule-II (SUCRA = 76%) was one of the higher-ranking interventions for reducing Caspase-3. In addition, high-dose Ming-Mu-Wu-Zi, high-dose Qu-Yu-Tong-Luo prescription, and the high-dose Bu-Yang-Huan-Wu decoction tend to demonstrate superior efficacy in neuroprotective outcomes compared to other treatments. These results indicate that TCM formulas require threshold concentrations to fully inhibit apoptosis damage and enhance retinal recovery (Jin et al., 2013). This dose-efficacy relationship not only strengthens the biological plausibility of our findings but also has direct implications for designing future preclinical and clinical studies, emphasizing the importance of optimizing dosage. However, the highest SUCRA values for certain indicators remain relatively low. For example, the BDNF network analysis was limited to data from only four treatments and lacked both direct and indirect comparisons. This limitation introduces uncertainty into the ranking results. Therefore, while SUCRA provides a preferable ranking reference, these specific results should be considered preliminary and hypothesis-generating. The clinical significance should not be overinterpreted without additional evidence. Future studies utilizing larger, more densely connected networks are warranted to validate these rankings and reduce the associated uncertainty.

Assessing publication bias is critical for interpreting pooled results, as selective publication remains an inherent limitation of evidence synthesis. In this study, Begg’s and Egger’s test indicated the presence of publication bias in the ERG-a wave and ERG-b wave outcomes. The observed publication bias for these indicators may stem from several factors, such as selective reporting of positive results, methodological heterogeneity across studies, or insufficient sample sizes, which could compromise the precision and reliability of the pooled estimates. Trim and fill analysis further demonstrated that incorporating three and four additional studies for ERG-a wave and ERG-b wave, respectively, could mitigate publication bias for these metrics. Therefore, the results of ERG-a and ERG-b waves should be interpreted with caution. Nevertheless, the retinal structure and functional parameters, including the RGC counts, and retinal thickness, showed no evidence of publication bias and significantly improved. Additionally, core outcomes such as neural growth, glial activation, oxidative stress and apoptotic indicators remained robust and unbiased. Therefore, this specific bias does not invalidate the study’s overarching neuroprotective conclusion, as the findings do not depend solely on this single outcome.

Although this meta-analysis provides preliminary evidence for the neuroprotective effects of TCM formulas, several limitations should be considered when the findings are interpreted. First, all the included studies were preclinical animal experiments, and the generalizability of our findings is further constrained by risks of bias in terms of randomization procedures, implementation of blinding, and outcome assessment, which may potentially overestimate the treatment effects. Second, the included TCM formulas exhibited variations in compositions, dosages, preparation methods, and routes of administration, which may have influenced the pooled results. Third, some network meta-analyses include a small number of studies, which may limit the stability of the results. Fourth, the association between these shared compounds and the neuroprotective effects was primarily based on existing literature and database analyses. The precise causal relationship between these shared compounds and the formulas’ overall therapeutic effects requires further elucidation. Therefore, these findings should be interpreted as preliminary evidence to guide future research rather than as conclusive results. The extrapolation of these results to clinical practice requires caution. Future preclinical studies with rigorous experimental designs are needed to address these limitations and enhance translational relevance.

## 5 Conclusion

This study offers exploratory and preliminary evidence that the included TCM formulas might exert neuroprotective effects on animal models of RDDs by promoting neurotrophic factors, inhibiting glial activation, reducing oxidative stress, and suppressing apoptosis. The definition of the compositions and identification of the bioactive compounds for these TCM formulas offer a preliminary pharmacological basis for their neuroprotective effects. Moving forwards, more high-quality preclinical studies on TCM formulas will be essential to validate their neuroprotective effects and translate them into clinically actionable strategies for treating RDDs.

## Data Availability

The original contributions presented in the study are included in the article/Supplementary Material, further inquiries can be directed to the corresponding author.

## Abbreviations

RDDs, Retinal degenerative diseases; AMD, Age-related macular degeneration; BDNF, Brain-derived neurotrophic factor; CNTF, Ciliary Neurotrophic Factor; GFAP, Glial fibrillary acidic protein; SOD, Superoxide dismutase; Caspase-3, Cysteine-dependent aspartate-specific protease 3; TCM, Traditional Chinese Medicine; RGC, Retinal ganglion cell; NMA, Network meta-analysis; SUCRA, Surface under the cumulative ranking curve; SD, Sprague-Dawley; DR, Diabetic retinopathy; MD, Mean difference; SMD, Standardized mean difference; MPNS, Medicinal Plant Names Services.
