# Supplementary material for: Neuroprotective effects of traditional Chinese medicine formulas in animal models of retinal degenerative diseases: a systematic review and meta-analysis
Source: Front Pharmacol. 2026 Jan 7;16:1695150. doi: 10.3389/fphar.2025.1695150 (PMC12819594; doi:10.3389/fphar.2025.1695150)
Supplement: Supplementary file 3 [file Supplementaryfile1.docx]

**Data sheet S1. Search Strategies in eight databases**

**Table 1.** Search strategy in PubMed database

| **Number** | **Search terms** | | **Results** |  |
| --- | --- | --- | --- | --- |
| #1 | | ("Retinal Degeneration"[MeSH] OR "Retinal Degenerative Diseases"[Title/Abstract] OR "Retinal Degeneration"[Title/Abstract] OR "Retinal Dystrophy"[Title/Abstract] OR "Retinal Disease"[Title/Abstract]) | 63,346 |  |
| #2 | | ("Brain-Derived Neurotrophic Factor"[MeSH] OR "BDNF"[Title/Abstract] OR "Brain Derived Neurotrophic Factor"[Title/Abstract]) | 36,499 |  |
| #3 | | #1 AND #2 | 142 |  |
| #4 | | ("Ciliary Neurotrophic Factor"[MeSH] OR "CNTF"[Title/Abstract] OR "Ciliary Neurotrophic Factor"[Title/Abstract]) | 3,034 |  |
| #5 | | #1 AND #4 | 137 |  |
| #6 | | ("Glial Fibrillary Acidic Protein"[MeSH] OR "GFAP"[Title/Abstract] OR "Glial Fibrillary Acidic Protein"[Title/Abstract]) | 36,165 |  |
| #7 | | #1 AND #6 | 454 |  |
| #8 | | ("Superoxide Dismutase"[MeSH] OR "SOD"[Title/Abstract] OR "Superoxide Dismutase"[Title/Abstract]) | 132,357 |  |
| #9 | | #1 AND #8 | 214 |  |
| #10 | | ("Caspase-3"[MeSH]OR"Caspase-3"[Title/Abstract] OR "CASP3"[Title/Abstract]) | 93,387 |  |
| #11 | | #1 AND #10 | 317 |  |
| #12 | | ("Medicine, Chinese Traditional"[MeSH] OR "Chinese Herbal Formula"[Title/Abstract] OR "Chinese Herbal Medicine"[Title/Abstract] OR "Traditional Chinese Medicine"[Title/Abstract] OR "Herbal Formula"[Title/Abstract]) | 63,602 |  |
| #13 | | (#3 AND #12) OR (#5 AND #12) OR (#7 AND #12) OR (#9 AND #12) OR (#11 AND #12) | 5 |  |

**Table 2.** Search strategy in Web of Science.

| Number | Search term | Results |
| --- | --- | --- |
| #1 | (TS=("retinal degenerat*" OR "retinal dystroph*" OR "macular degenerat*")) | 38,641 |
| #2 | (TS=("brain derived neurotrophic factor" OR "BDNF" OR "ciliary neurotrophic factor" OR "CNTF" OR "glial fibrillary acidic protein" OR "GFAP" OR "superoxide dismutase" OR "SOD" OR "caspase 3" OR "caspase-3" OR "CASP3")) | 315,341 |
| #3 | (TS=("Chinese herbal*" OR "traditional Chinese medicine*" OR "TCM")) | 55,541 |
| #4 | #1 AND #2 AND #3 | 8 |

**Table 3.** Search strategy in Cochrane database.

| Number | Search term | Results |
| --- | --- | --- |
| #1 | MeSH descriptor: [Retinal Degeneration] explode all trees | 3,797 |
| #2 | MeSH descriptor: [Brain-Derived Neurotrophic Factor] explode all trees | 666 |
| #3 | MeSH descriptor: [Ciliary Neurotrophic Factor] explode all trees | 24 |
| #4 | MeSH descriptor: [Glial Fibrillary Acidic Protein] explode all trees | 56 |
| #5 | MeSH descriptor: [Superoxide dismutase] explode all trees | 1,030 |
| #6 | MeSH descriptor: [Caspase-3] explode all trees | 89 |
| #7 | (Chinese herbal formula):ti,ab,kw | 753 |
| #8 | #1 AND (#2 OR #3 OR #4 OR #5 #6) AND #7 | 0 |

**Table 4.** Search strategy in Embase database.

| Number | Search term | Results |
| --- | --- | --- |
| #1 | 'retina degeneration'/exp OR ('retinal degeneration*' OR 'retinal dystroph*' OR 'retin* degenerat*') | 95,489 |
| #2 | 'brain derived neurotrophic factor'/exp OR ('BDNF' OR 'brain-derived neurotrophic factor*') | 64,722 |
| #3 | 'ciliary neurotrophic factor'/exp OR ('CNTF' OR 'ciliary neurotrophic factor*') | 5,668 |
| #4 | 'glial fibrillary acidic protein'/exp OR ('GFAP' OR 'glial fibrillary acidic protein*') | 58,483 |
| #5 | 'superoxide dismutase'/exp OR ('SOD' OR 'superoxide dismutase') | 197,541 |
| #6 | 'caspase 3'/exp OR ('caspase-3' OR 'caspase 3') | 159,930 |
| #7 | 'Chinese drug'/exp OR 'herbal medicine'/exp OR ('Chinese herbal*' OR 'TCM' OR 'herbal formula*') | 136,715 |
| #8 | #2 OR #3 OR #4 OR #5 OR #6 | 462,354 |
| #9 | #1 AND #7 AND #8 | 31 |

**Table 5.** Search strategy in CNKI database.

| Number | Search term | Results |
| --- | --- | --- |
| #1 | 视网膜[主题] OR视网膜退行性疾病[主题] | 90,079 |
| #2 | BDNF[主题]OR brain-derived neurotrophic factor[主题] | 17,161 |
| #3 | #1 AND #2 | 323 |
| #4 | CNTF[主题] OR ciliary neurotrophic factor[主题] | 1,179 |
| #5 | #1 AND #4 | 191 |
| #6 | GFAP[主题] OR glial fibrillary acidic protein[主题] | 10,550 |
| #7 | #1 AND #6 | 457 |
| #8 | SOD[主题] OR superoxide dismutase[主题] | 131,408 |
| #9 | #1 AND #8 | 885 |
| #10 | Caspase-3[主题] | 11,829 |
| #11 | #1 AND #10 | 163 |
| #12 | 中药复方 | 21,127 |
| #13 | #12 AND (#3 OR #5 OR #7 OR #9 OR #11) | 1864 |

**Table 6.** Search strategy in Wan Fang database.

| Number | Search term | Results |
| --- | --- | --- |
| #1 | 主题：(视网膜) OR主题：(视网膜退行性疾病) | 147,881 |
| #2 | 主题：(BDNF) OR主题：(CNTF) OR 主题：(GFAP) OR 主题: (SOD) OR 主题: (Caspase-3) | 302,691 |
| #3 | [主题:(中医药) or 主题:(中药复方) or 主题:(方剂)](https://ras.cdutcm.edu.cn:7080/s/cn/com/wanfangdata/s/G.https/advanced-search/paper?q=%E4%B8%BB%E9%A2%98%3A(%E4%B8%AD%E5%8C%BB%E8%8D%AF)%20or%20%E4%B8%BB%E9%A2%98%3A(%E4%B8%AD%E8%8D%AF%E5%A4%8D%E6%96%B9)%20or%20%E4%B8%BB%E9%A2%98%3A(%E6%96%B9%E5%89%82)&searchtype=expert&type=%5b%22periodical%22,%22thesis%22,%22conference%22%5d&chineseEnglishExpand=true) | 452,859 |
| #4 | #1 AND #2 AND #3 | 154 |

**Table 7.** Search strategy in VIP Medical Information database.

| Number | Search term | Results |
| --- | --- | --- |
| #1 | [(题名或关键词=视网膜 OR 题名或关键词=视网膜退行性疾病)](https://ras.cdutcm.edu.cn:7080/s/com/cqvip/qikan/G.http/Qikan/search/index?LngMySearHistoryIdGuid=568ee5a6-182a-44c6-9584-555eb08126c5&from=Qikan_Article_History) | 61,047 |
| #2 | [((((题名或关键词=BDNF OR 题名或关键词=CNTF) OR 题名或关键词=GFAP) OR 任意字段=SOD) OR (任意字段=Caspase AND ( NOT 任意字段=3)))](https://ras.cdutcm.edu.cn:7080/s/com/cqvip/qikan/G.http/Qikan/search/index?LngMySearHistoryIdGuid=a2f984fd-02f9-40ab-9b9d-ea036043ec90&from=Qikan_Article_History) | 106,774 |
| #3 | [((题名或关键词=中医药 OR 题名或关键词=中药复方) OR 题名或关键词=方剂)](https://ras.cdutcm.edu.cn:7080/s/com/cqvip/qikan/G.http/Qikan/search/index?LngMySearHistoryIdGuid=66b43ab7-bd2a-4921-bef9-6ae052dae829&from=Qikan_Article_History) | 308,396 |
| #4 | #1 AND #2 AND #3 | 12 |

**Table 8.** Search strategy in CBM database.

| Number | Search term | Results |
| --- | --- | --- |
| #1 | (视网膜) OR 视网膜退行性疾病 | 15954 |
| #2 | ((((BDNF) OR CNTF) OR GFAP) OR SOD) OR Caspase-3 | 8667 |
| #3 | (中医药) OR 中药复方) OR 方剂 | 5408 |
| #4 | #1 AND #2 AND #3 | 10 |
|  |  |  |

**
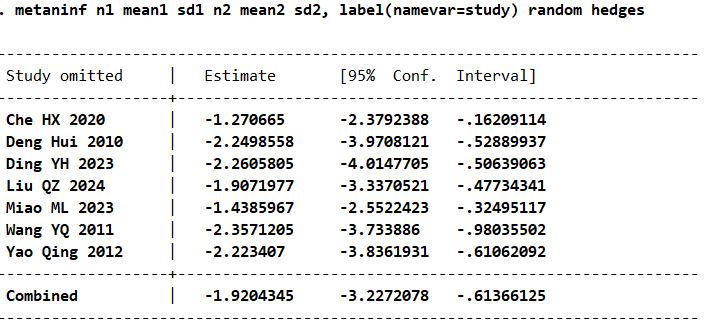
D****ata sheet S2. Sensitivity analyses in GFAP, CNTF, RGC counts, retinal thickness**

Figure 1:sensitivity analysis in GFAP


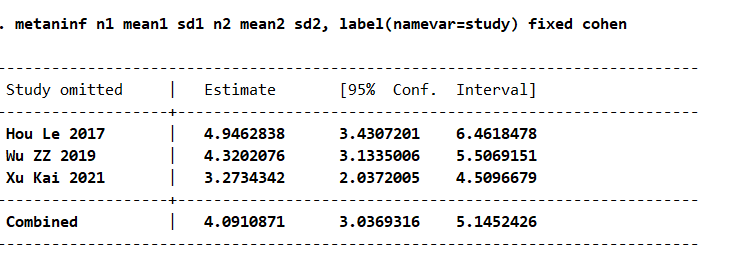
**
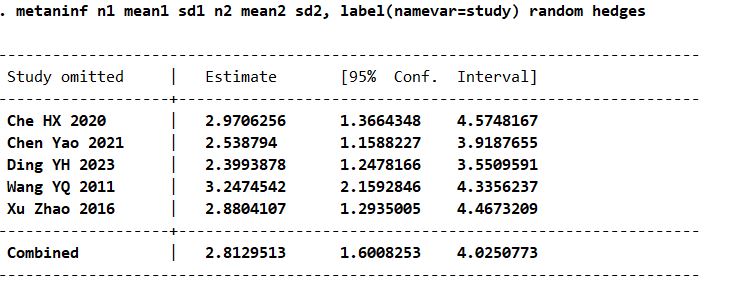
**

Figure 2 : sensitivity analysis in CNTF

Figure 3 : sensitivity analysis in RGC counts

**
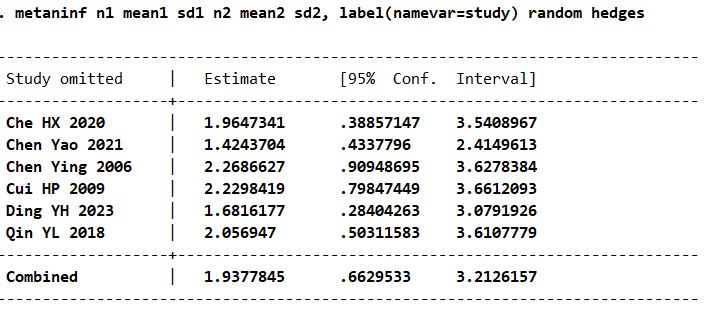
**Figure 4 :Sensitivity analyses of retinal thickness

**
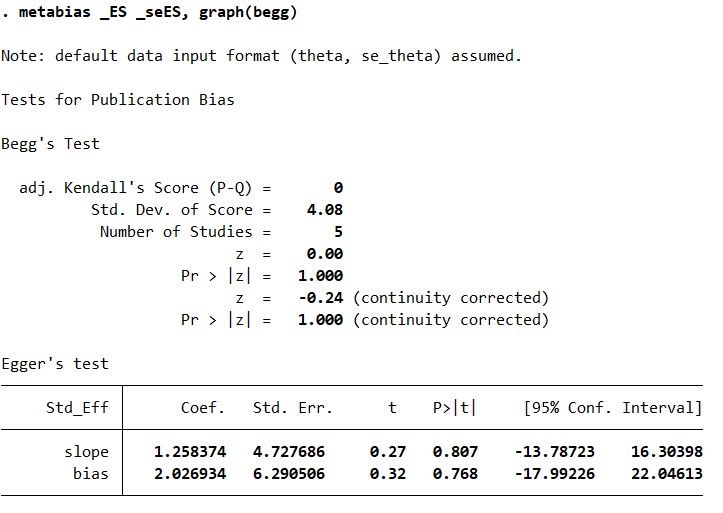
D****ata sheet S3. Publication analyses of included indicators**

Figure 1: publication analysis in RGC counts

**
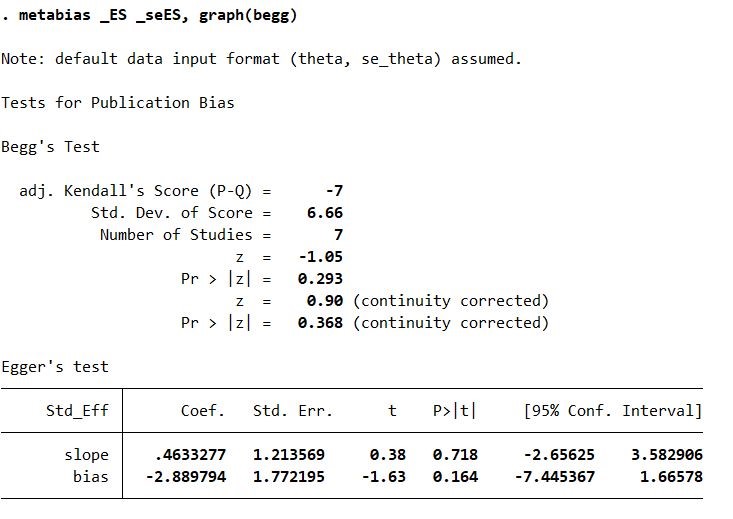
**

**
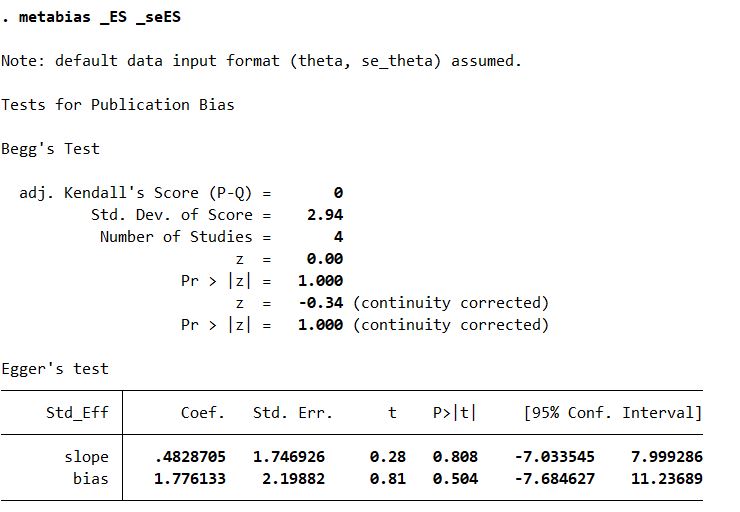
**Figure 2: publication analysis in GFAP

Figure 3: publication analysis in BDNF

**
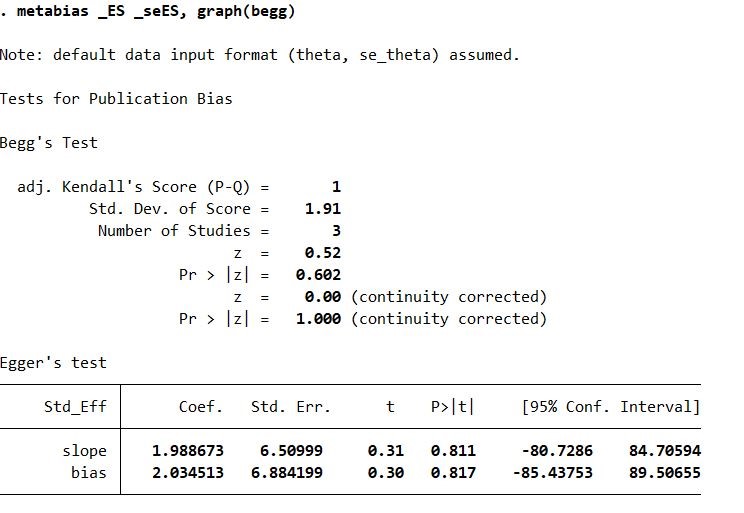
**Figure 4: publication analysis in CNTF

**
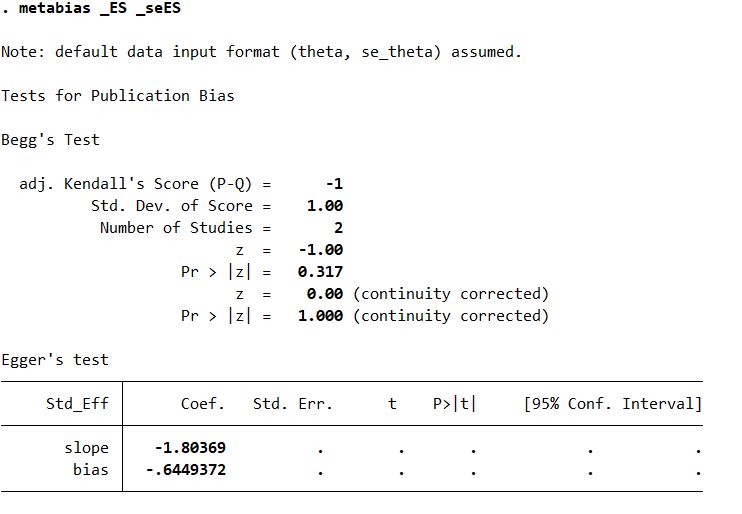
**Figure 5: publication analysis in caspase-3

**
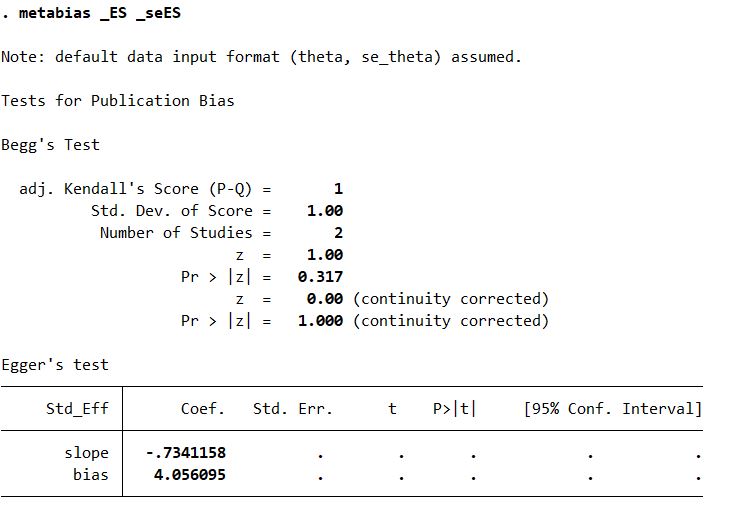

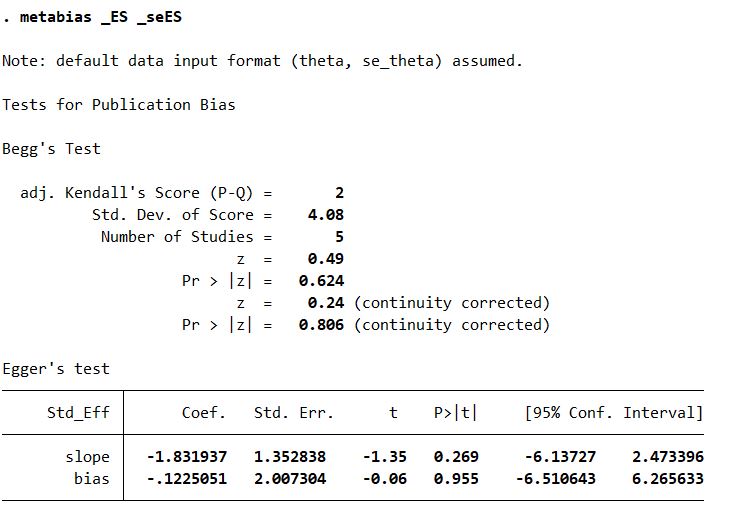
**Figure 6: publication analysis in retinal apoptotic cell counts

Figure 7: publication analysis in retinal SOD

**
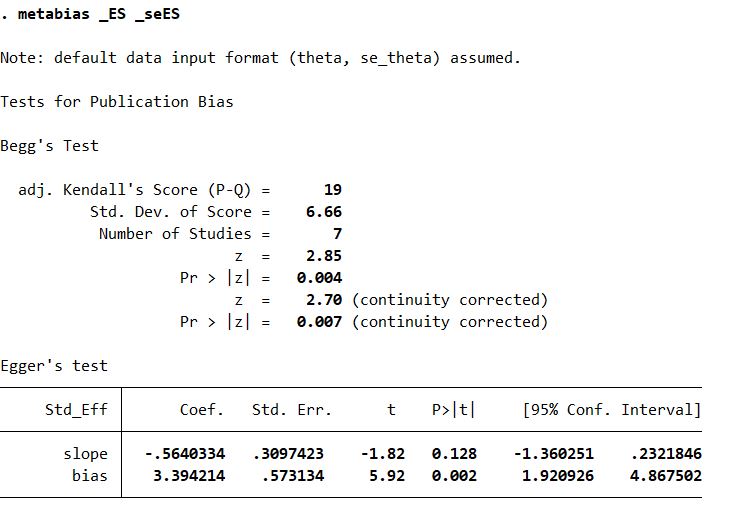
**Figure 8: publication analysis in retinal ERG-a wave

Figure 9: publication analysis in retinal ERG-a wave **
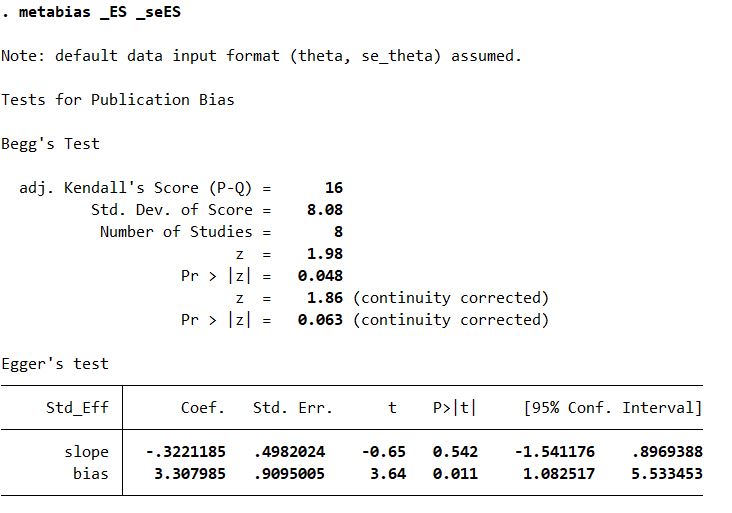
**

Figure 10: publication analysis in retinal ERG-a wave **
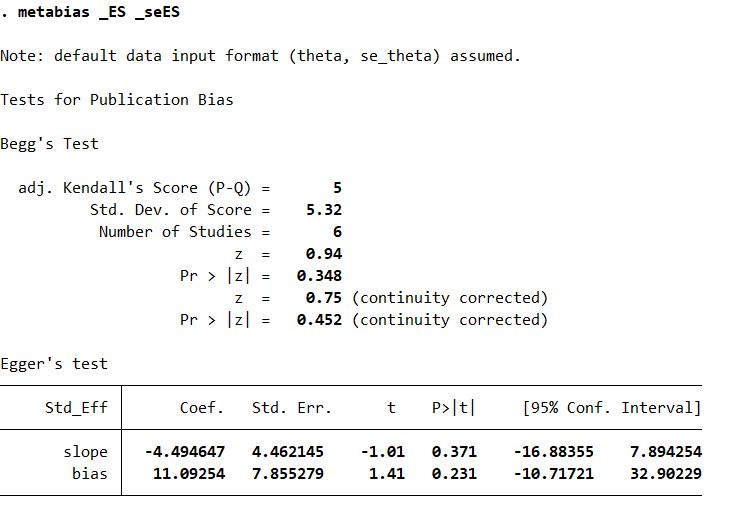
**

**Data sheet S4. Trim and fill analyses of ERG-a wave and ERG-b wave**


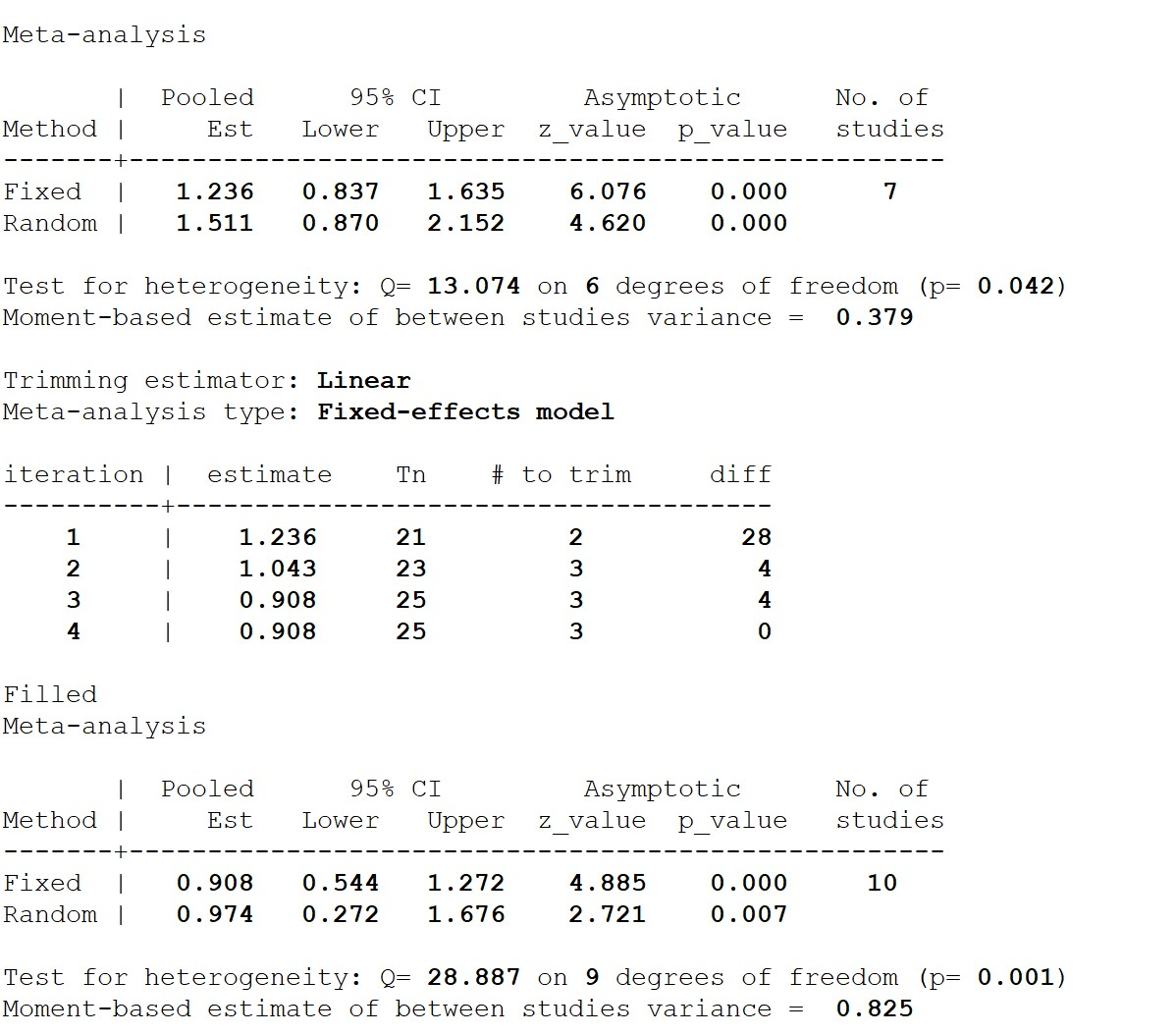


Figure 1: The results of trim and fill analysis of ERG-a wave


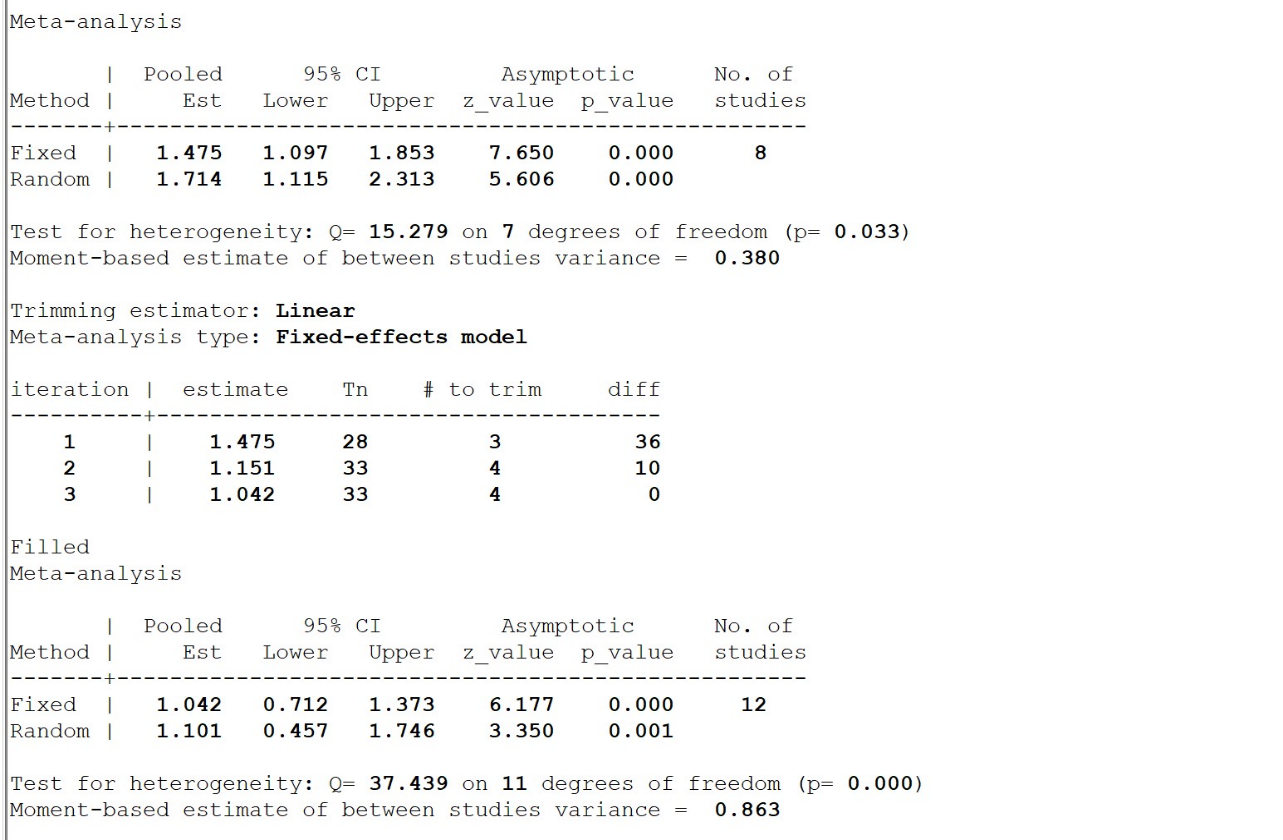
Figure 2: The filed funnel plot in trim and fill analysis of ERG-a wave

Figure 3: The filed funnel plot in trim and fill analysis of ERG-b wave

Figure 4: The filed funnel plot in trim and fill analysis of ERG-b wave

**
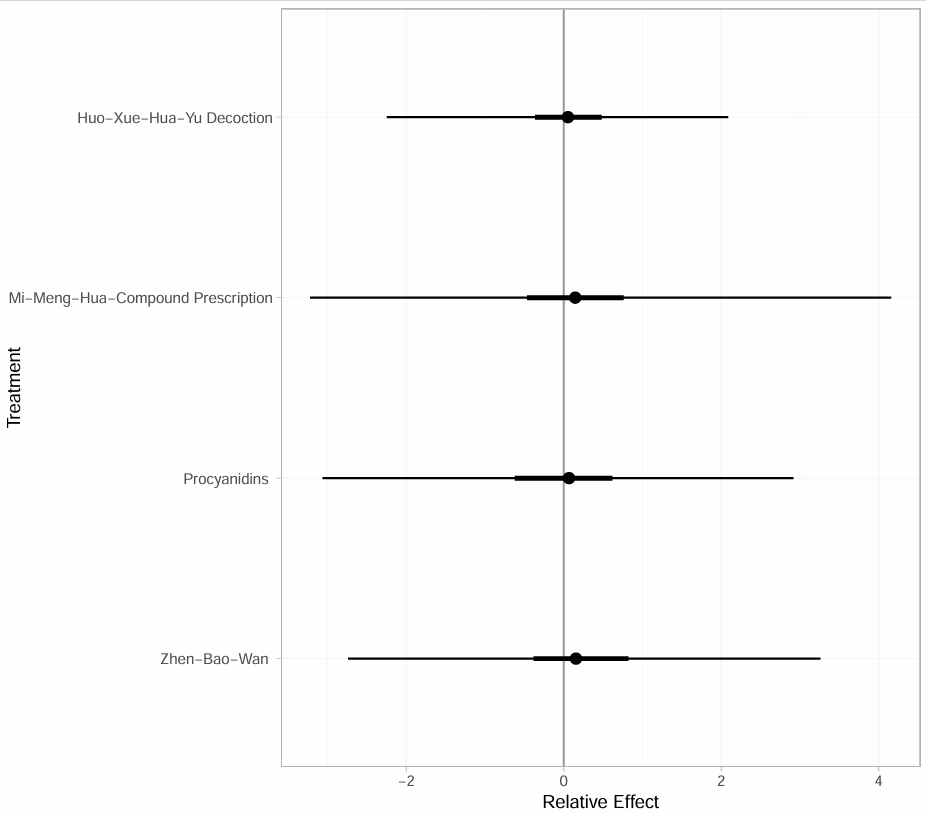
D****ata sheet S5:Forest figures compared with the model group in NMA analyses**

Figure 1: BDNF forest figure

**
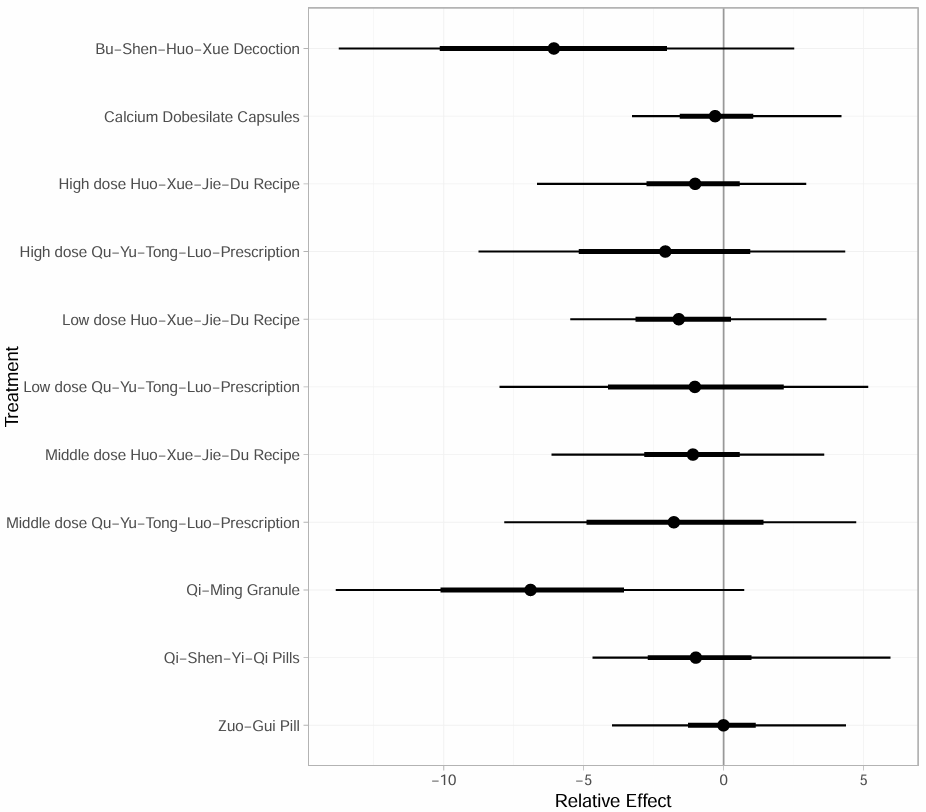
** Figure 2: GFAP forest figure


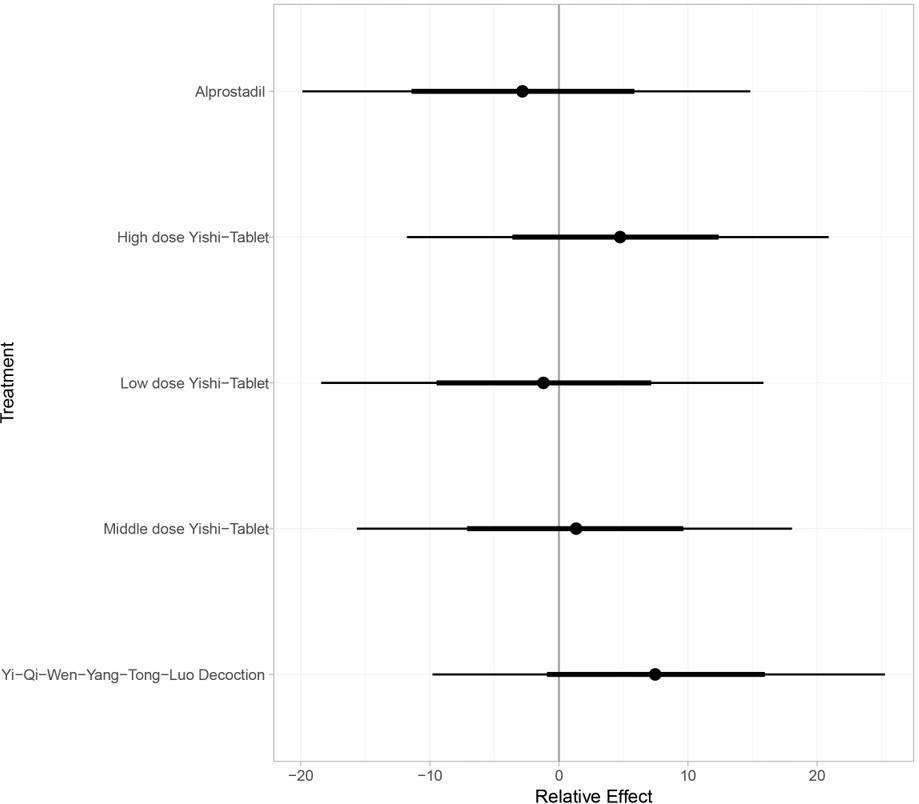


Figure 3: SOD forest figure

**
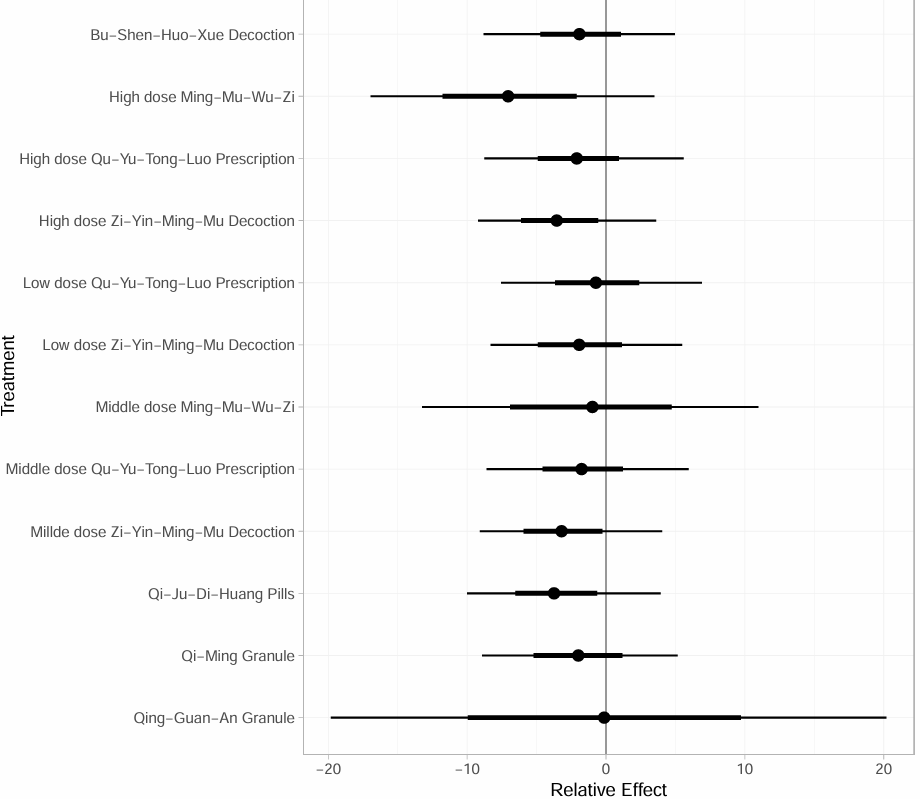
**

Figure 4: retinal apoptotic cell counts forest figure

**
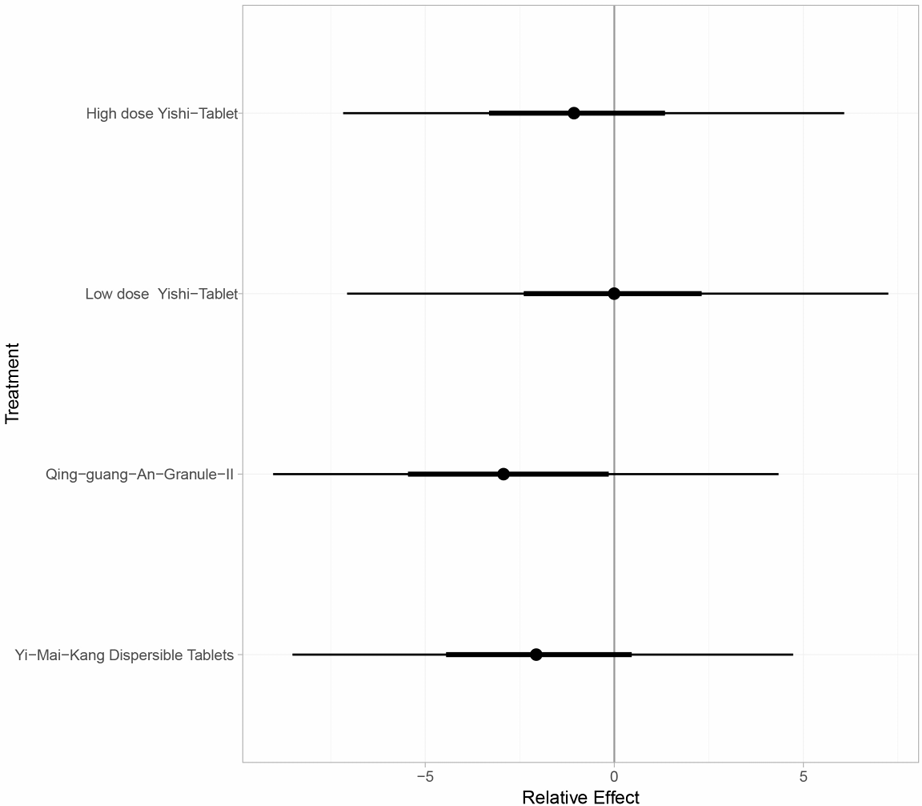
**

Figure 5: caspase-3 forest figure

**
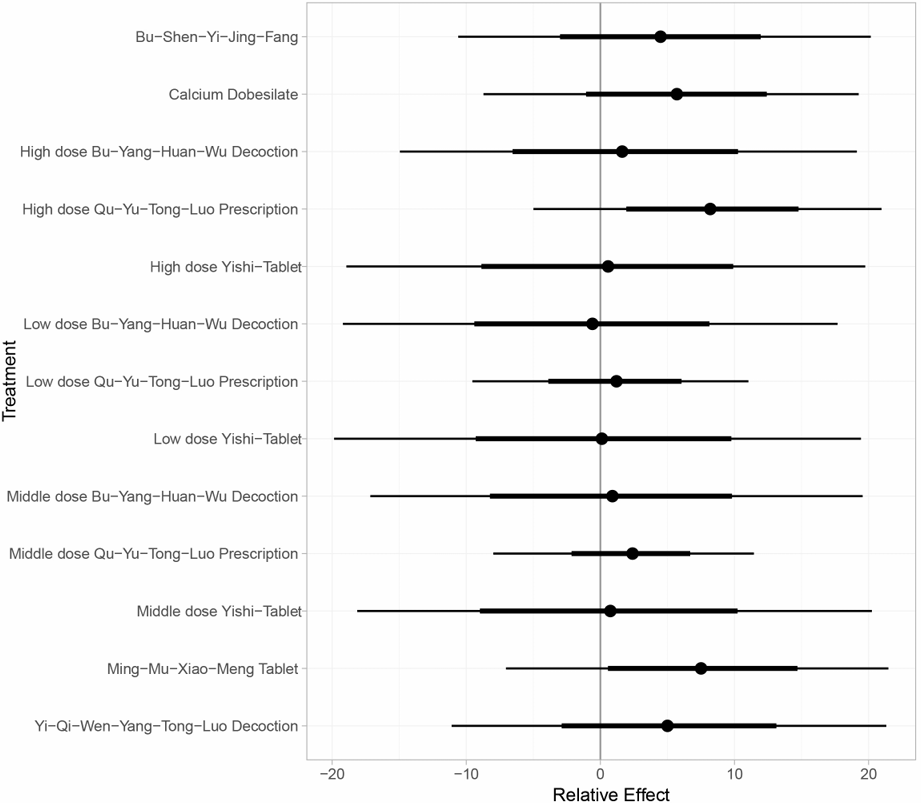
**

Figure 6: ERG-a wave forest figure

**
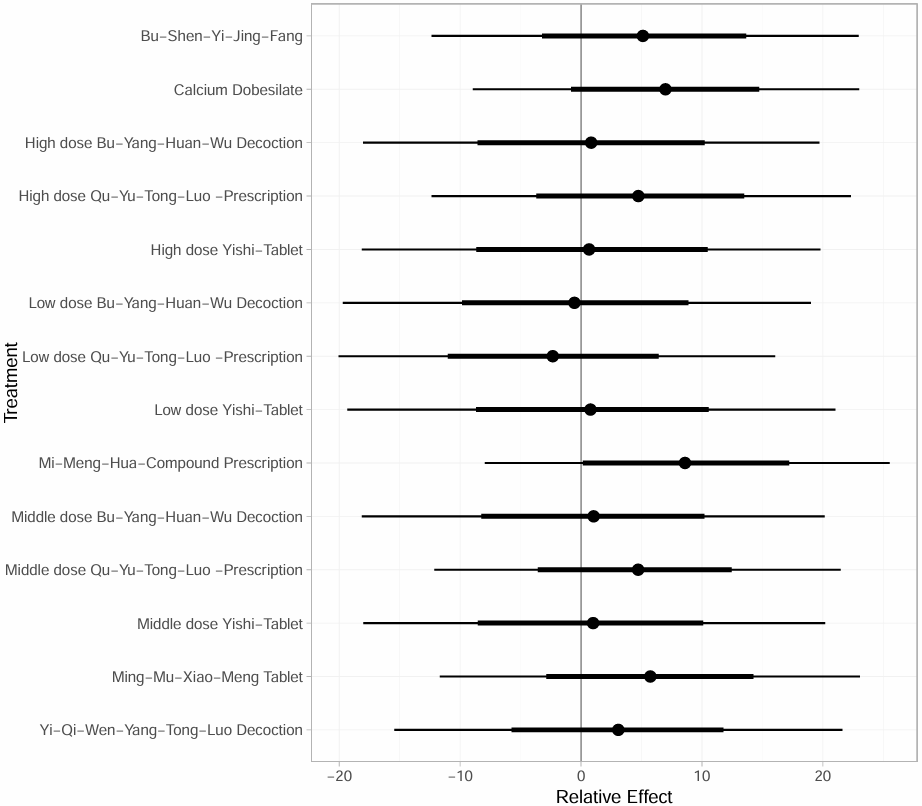
**

Figure 7: ERG-b wave forest figure

**
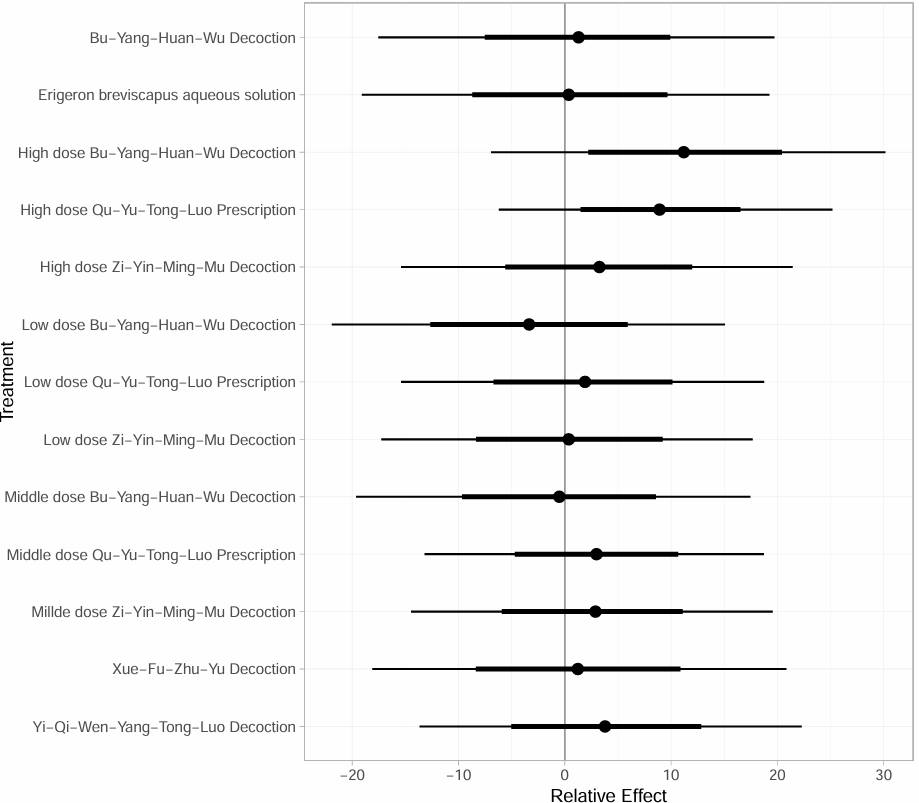
**

Figure 8: retinal thickness forest figure

**
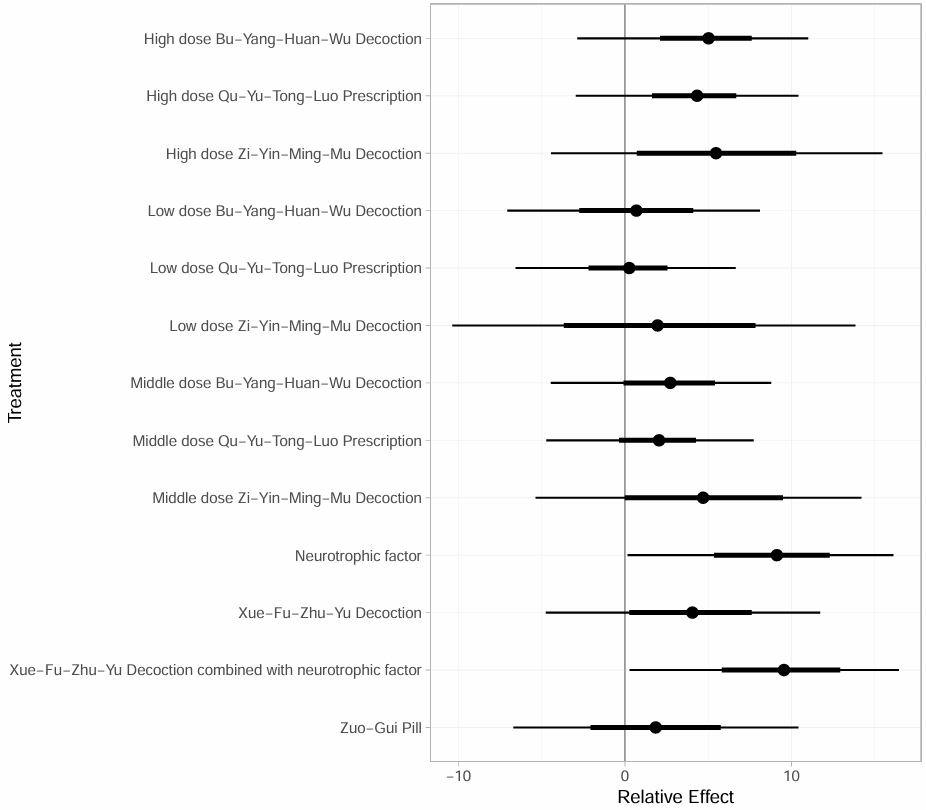
**

Figure 9: RGC counts forest figure

**Data sheet S6. Results of SUCRA analyses in NMA analyses**


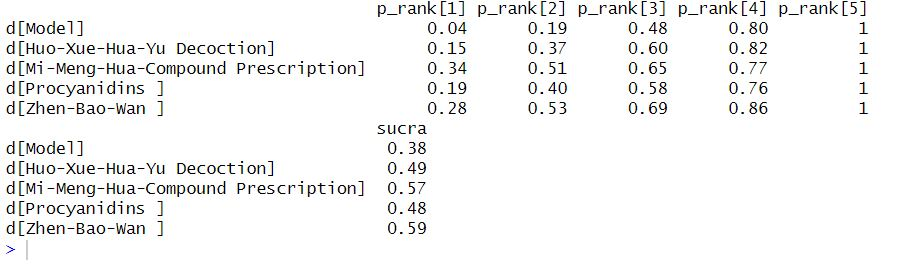


Figure 1: BDNF SUCRA ranking


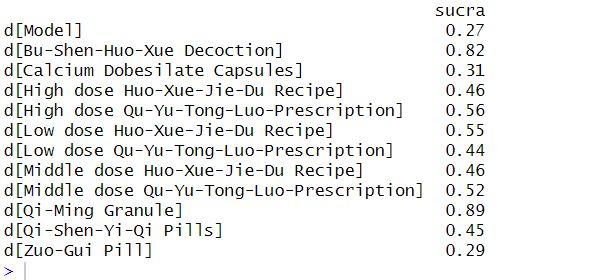


Figure 2: GFAP ranking


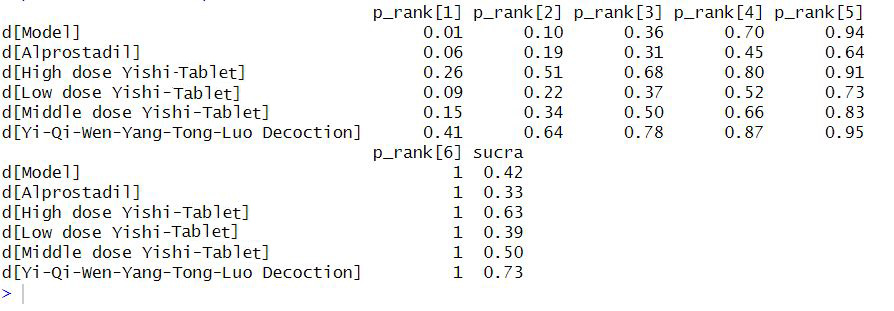


Figure 3: SOD ranking


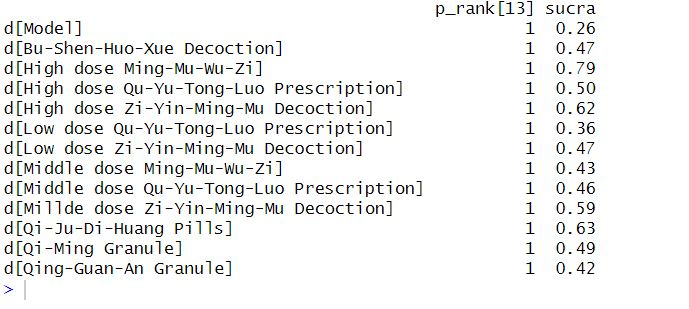
Figure 4: retinal apoptotic cell counts ranking

**
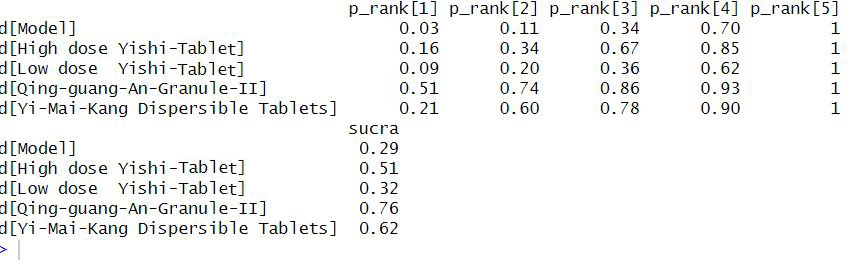
**


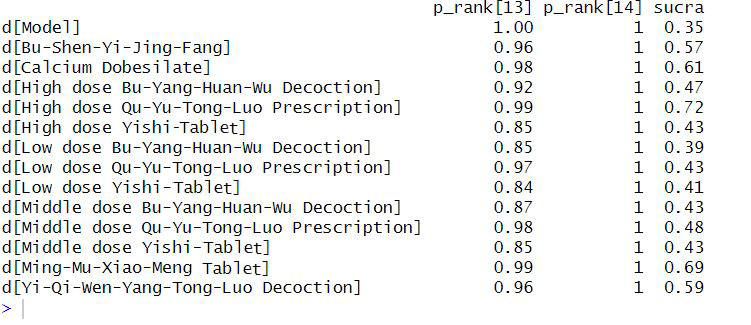
Figure 5: caspase-3 ranking

Figure 6: ERG-a wave ranking


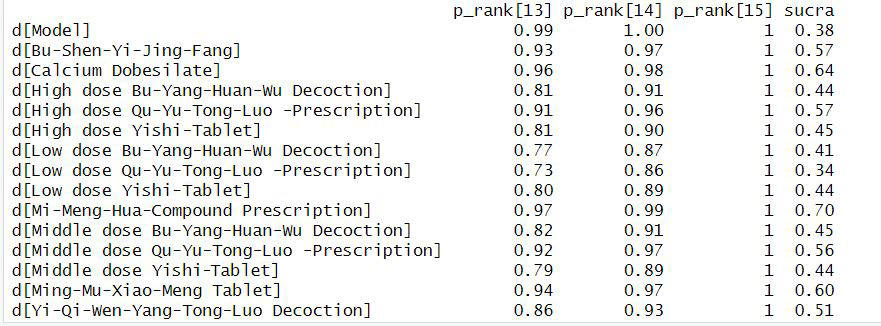

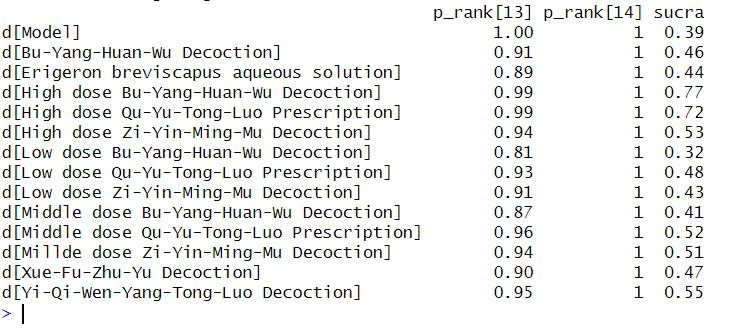
 Figure 7: ERG-b wave ranking


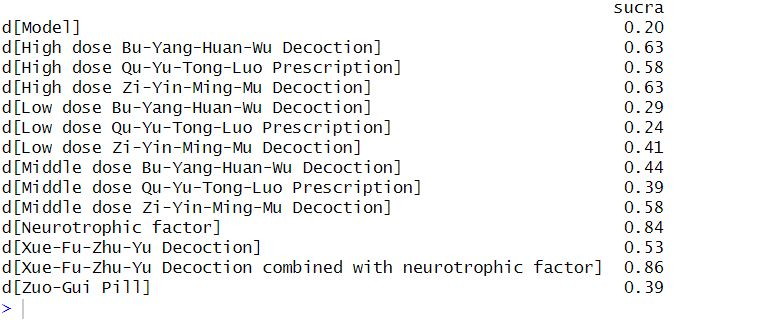
Figure 8: retinal thickness ranking

Figure 9: RGC counts ranking

**Data sheet S7.** **Results of consistency assumption**


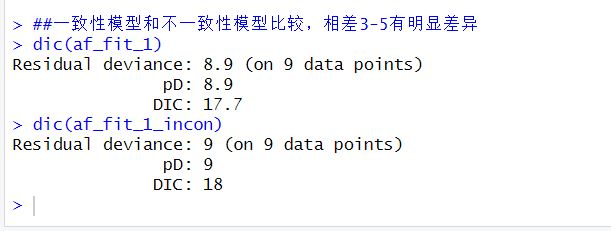


Figure 1: BDNF


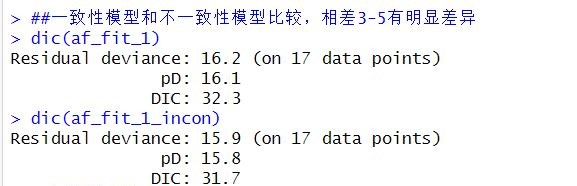


Figure 2: GFAP


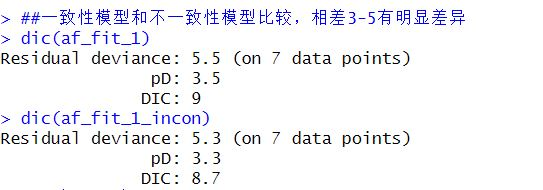


Figure 3: SOD


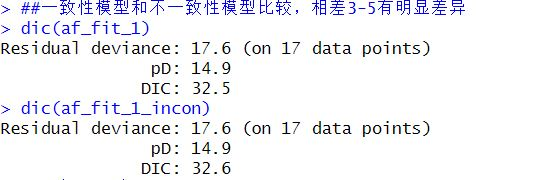


Figure 4: retinal apoptotic cell counts


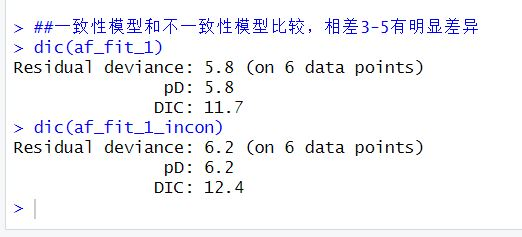


Figure 5: caspase-3


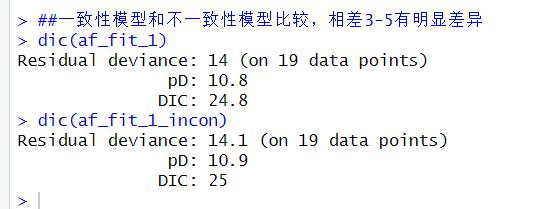


Figure 6: ERG-a wave


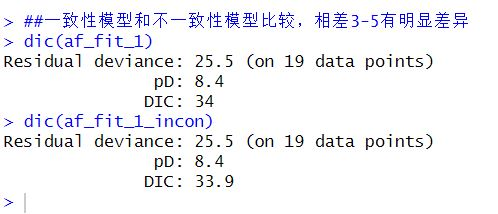

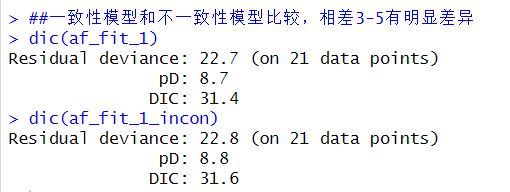
Figure 7: ERG-b wave


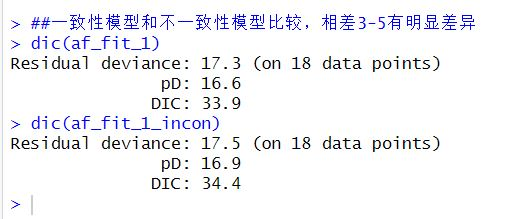
Figure 8: retinal thickness

Figure 9: RGC counts
